# Less Exploited GPCRs in Precision Medicine: Targets for Molecular Imaging and Theranostics

**DOI:** 10.3390/molecules24010049

**Published:** 2018-12-23

**Authors:** João Franco Machado, Rúben D. Silva, Rita Melo, João D. G. Correia

**Affiliations:** 1 Centro de Ciências e Tecnologias Nucleares, Instituto Superior Técnico, Universidade de Lisboa, CTN, Estrada Nacional 10 (km 139,7), 2695-066 Bobadela LRS, Portugal; joaomachado@campus.ul.pt (J.F.M.); rdm.silva@campus.fct.unl.pt (R.D.S.); ritamelo@ctn.tecnico.ulisboa.pt (R.M.); 2 Centro de Química Estrutural, Faculdade de Ciências, Universidade de Lisboa, 1749-016 Lisboa, Portugal; 3 Center for Neuroscience and Cell Biology; Rua Larga, Faculdade de Medicina, Polo I, 1ºandar, Universidade de Coimbra, 3004-504 Coimbra, Portugal

**Keywords:** frizzled receptor (FZD), ghrelin receptor (GHSR-1a), G protein-coupled estrogen receptor (GPER), molecular imaging, Sphingosine-1-phosphate receptor (S1PR), theranostics

## Abstract

Precision medicine relies on individually tailored therapeutic intervention taking into account individual variability. It is strongly dependent on the availability of target-specific drugs and/or imaging agents that recognize molecular targets and patient-specific disease mechanisms. The most sensitive molecular imaging modalities, Single Photon Emission Computed Tomography (SPECT) and Positron Emission Tomography (PET), rely on the interaction between an imaging radioprobe and a target. Moreover, the use of target-specific molecular tools for both diagnostics and therapy, theranostic agents, represent an established methodology in nuclear medicine that is assuming an increasingly important role in precision medicine. The design of innovative imaging and/or theranostic agents is key for further accomplishments in the field. G-protein-coupled receptors (GPCRs), apart from being highly relevant drug targets, have also been largely exploited as molecular targets for non-invasive imaging and/or systemic radiotherapy of various diseases. Herein, we will discuss recent efforts towards the development of innovative imaging and/or theranostic agents targeting selected emergent GPCRs, namely the Frizzled receptor (FZD), Ghrelin receptor (GHSR-1a), G protein-coupled estrogen receptor (GPER), and Sphingosine-1-phosphate receptor (S1PR). The pharmacological and clinical relevance will be highlighted, giving particular attention to the studies on the synthesis and characterization of targeted molecular imaging agents, biological evaluation, and potential clinical applications in oncology and non-oncology diseases. Whenever relevant, supporting computational studies will be also discussed.

## 1. Introduction

Precision medicine, sometimes named personalized medicine, is a fast-growing approach for disease treatment, which, in general terms, aims at delivering an “appropriate dose of a right drug to a right patient” [1,2,3,4]. Unlike traditional ‘one-size-fits-all’ therapeutic approaches, precision medicine relies on individually tailored therapeutic intervention, taking into account individual variability. Thus, treatments are targeted on the basis of genetic, phenotypic, or psychosocial characteristics that differentiate patients [1,2,3,4]. In this way, it is expected to improve the therapeutic outcome, maximizing treatment efficacy with minimization of adverse side effects and toxicity. This strategy is strongly dependent on the availability of target-specific drugs and/or imaging agents that recognize, with high affinity, specific molecular targets and patient-specific mechanisms related to a disease [5,6]. Within this context, noninvasive medical imaging is of paramount importance, namely in terms of screening, early diagnosis, treatment follow up, and assessing likelihood of disease recurrence [7,8,9]. Imaging contributes to identifying and stratifying patients with identical disease characteristics and share similar treatment response and prognosis. Molecular imaging, in particular, defined as the in vivo characterization and measurement of biological processes at the cellular and molecular level, allows for imaging of the expression and activity of specific target molecules (e.g., enzymes and cell surface receptors) as well as biological processes (e.g., angiogenesis or apoptosis) [10]. Once validated, it is an efficient tool for assessing critical issues, namely early detection of disease, identifying metabolic alterations before anatomical signs appear, diagnosis, risk-stratification, and assessing pharmacokinetics and pharmacodynamics of drugs [11]. The nuclear imaging modalities Single Photon Emission Computed Tomography (SPECT) and Positron Emission Tomography (PET) are the most sensitive imaging modalities currently available [12,13,14], but there is also a great effort worldwide to bringing Magnetic Resonance Imaging (MRI) or other modalities into the molecular imaging arena [15,16]. The in vivo noninvasive visualization of a specific biological target relies on its interaction with a specific imaging probe [13,17,18,19]. Moreover, the use of target-specific molecular tools both for diagnostics and therapy, named theranostic agents, is a well-established approach in nuclear medicine, with an increasingly important role in precision medicine. This approach takes advantage of the same or similar molecular entities, which are either radiolabeled differently or given in different dosages [20,21,22,23,24]. In cancer treatment, imaging of the radiolabeled molecule can be used as a biomarker for the drug to predict patient response as well as for therapy follow-up with conventional chemotherapy agents. Within the strict context of nuclear medicine, the theranostic approach makes use of the same targeting molecule which is radiolabeled with a beta-emitting radionuclide (e.g., ^177^Lu or ^90^Y) for therapy or with a gamma- (e.g., ^111^In) or positron-emiting (e.g., ^68^Ga) radionuclide for imaging and to guide follow-on systemic radiotherapy. However, it is important to mention that the two types of agents must fulfill different requisites and not all imaging agents can be converted into efficient agents for systemic therapy. For the sake of example, in the case of an imaging agent, ideally, high tumor accumulation should last only until image is acquired, whereas in the case of systemic radiotherapy prolonged tumor retention is desired in order to provide a sufficient radiation dose to the tumor. Another relevant aspect in theranostics is the use of quantitative imaging to provide patient-specific dosimetry to guide subsequent radionuclide therapy. Imaging dosimetry requirements are out of the scope of this review article, being the readers directed to recent articles on this key topic [25,26,27,28].

One of the deficiencies of these multidisciplinary research areas, which require comprehensive expertise from various fields (e.g., medicine, biology, chemistry, engineering, physics, among others), has been the lack of adequate ways to specifically and quantitatively visualize targets of interest and to interrogate biological events in patients. There is still a lack of imaging agents that can specifically probe molecular targets and biological activities [20,21,22,23]. Their identification is critical for early detection and risk-stratification of different diseases. The design of innovative imaging and/or theranostic agents are key for clinical application of molecular imaging and radionuclide therapy, and benefits precision medicine [11]. Within this context, G-protein-coupled receptors (GPCRs), apart from being intensively studied drug targets [29], have been also largely exploited as molecular targets for noninvasive imaging and/or systemic radiotherapy of various diseases. The most paradigmatic and clinically successful example is the use of somatostatin receptors (SSTRs)-targeted radiolabeled somatostatin analogs for imaging and peptide receptor radionuclide therapy of neuroendocrine tumors (NETs) [30]. A set of excellent reviews has surveyed the most relevant achievements in this field, namely SPECT and PET imaging [31,32,33,34,35,36,37,38,39], and radionuclide therapy [40,41], including theranostic applications [42,43]. Interestingly, it has been recently questioned whether neurotensin receptor (NTR), namely NTR1, could be the new SSTR [44]. Indeed, this receptor has been successfully targeted in vivo using radiolabeled molecules for diagnostic imaging and radionuclide therapy of NTR1-expressing tumors [45,46,47,48,49,50].

The gastrin-releasing peptide receptor (GRPR) has been also intensively explored as a target both for imaging [51,52,53,54,55,56,57,58,59] or radionuclide therapy [41,60,61,62,63,64,65] of prostate cancer and other GRPR-expressing cancers. For those applications, ^68^Ga-labeled specific tracers have a well-established diagnostic relevance and the clinical utility of specific radiolabeled peptides has been also clearly demonstrated.

Despite the thorough research efforts initially directed towards the development of radioactive peptides for targeting cholecystokinin 2 (CCK2)/gastrin receptor aiming at the detection/visualization or treatment of CCK2 receptor-expressing tumors [66], not many advances have been perceived in the past years. Such a situation, namely the low number of preclinical or clinical assays reported in the literature [67,68,69,70,71], has been partially ascribed to the in vivo instability of the targeting peptides [72,73,74,75]. Although not as extensively as the peptide receptors mentioned above, neuropeptide Y receptor subtypes 1 (NPYR1) and 2 (NPYR2) have also been studied as imaging targets for NPYR1-expressing tumors such as breast cancer, [76,77,78,79,80,81,82,83,84] and for brain imaging [85], respectively.

Another example of a less exploited GPCR for molecular imaging is the endothelin (ET) axis, which comprises the endothelin peptides (ET-1, ET-2, and ET-3) and their receptors, the endothelin receptor subtype A (ETA) and B (ETB). Besides its key physiological functions in particularly in the cardiovascular system, the ET axis also plays a major role in growth and progression of various tumors. To date, most of the efforts have been directed towards the development of radioactive receptor ligands for the visualization of endothelin receptor expression in vivo in cancer or in the heart [86,87,88,89,90,91,92].

The peptide receptor melanocortin type 1 receptor (MC1R) has also been studied as a potential target for diagnostic imaging and therapy of melanoma and metastases, using mainly radiolabeled peptides. A set of relevant articles, including reviews, have appeared in the last years [93,94,95,96,97,98,99,100,101,102]. However, as far as the authors are aware, no relevant human clinical applications have emerged.

The visualization of vasoactive intestinal peptide receptor (VPACR)-expressing tumors has been successfully attained using stable radiolabeled VIP derivatives and nuclear imaging techniques [103,104,105,106].

All the aminergic receptors are well-established drug targets, accounting for circa 70% of the GPCR drug targets with clinical relevance and impact [29]. Consequently, imaging of these receptors has been largely exploited, reaching a central role in the management of various diseases. Indeed, histamine [107,108,109,110,111,112], dopamine [16,113,114,115,116], and serotonin [117,118,119,120] receptors are important targets for in vivo nuclear imaging and target quantification in central nervous system disorders [121,122,123,124]. Likewise, noninvasive imaging of the postsynaptic cholinergic muscarinic receptors (mAChRs) would be potentially relevant for neurodegenerative diseases management and/or for the assessment of new target-specific drugs. However, accomplishments in this field have been scarce in the past decade, partially due to the low selectivity towards mAChRs shown by the probes developed [125,126,127,128,129]. A few PET probes for targeting specific adrenoreceptor subtypes have also been designed and assessed for cardiac [130,131,132] or brain [133,134,135] molecular imaging.

As regards the cannabinoid receptors, most of the research efforts aimed at the design of PET imaging probes to map and quantify these receptors in the living human brain, for drug development purposes or diagnostic of central nervous system (CNS) diseases [136,137,138,139,140,141,142]. Similarly, PET imaging of the adenosine receptor is potentially an additional tool to evaluate various aspects of neuroinflammatory and neurodegenerative diseases in vivo and has been the focus of excellent recent review articles addressing those issues [143,144,145,146,147].

Functional imaging of glutamate receptors with noninvasive techniques, mainly PET and SPECT [148,149,150] but recently MRI also [16], is being used to assess CNS disorders [121,123] and to select the appropriate dose of clinically relevant drug candidates targeting this receptor, among others. The importance of these discoveries has been highlighted by a set of excellent recent reviews that describe the progress in this area [16,148,149,150].

In the last few years, the chemokine receptor 4 (CXCR4) has also been successfully exploited in the clinical set as an imaging target for noninvasive monitoring of its expression in tumors as well as a target for receptor radionuclide therapy in cancer treatment [151,152,153,154,155,156,157,158,159].

Although not exhaustive, the general comments above give an overview of the majority of the GPCRs studied and established in the last decades as relevant molecular imaging targets both at the preclinical and clinical level. The issues addressed also comprise the use of receptor-imaging for theranostic applications as well as for drug development. Nevertheless, taking into account both the unmet needs in precision medicine and the increasing number of less exploited GPCRs that are currently targets of new drugs in clinical trials, we consider that imaging of these receptors holds great potential for application in drug development and theranostics of cancer and CNS diseases, among others. Herein, we will discuss recent efforts towards the design and development of innovative imaging and/or theranostic agents targeting selected emergent GPCRs, namely the Frizzled receptor (FZD), Ghrelin receptor (GHSR), G protein-coupled estrogen receptor (GPER), and Sphingosine-1-phosphate receptor (S1PR). The pharmacological and clinical relevance for targeting these receptors in oncology and non-oncology diseases will be highlighted, giving particular attention to the studies on the synthesis and characterization, biological evaluation and potential clinical applications. When available, relevant supporting computational studies will be also discussed in each section.

## 2. Frizzled Receptor (FZD)

The frizzled receptors (FZDs) constitute a family of GPCRs that comprises 10 FZD isoforms (FZD_1_–FZD_10_) in mammals [160]. FZD_1–10_ mediate their biological actions mainly through secreted lipoglycoproteins of the Wingless/Int-1 (WNT) family [160]. Interaction of FZDs with Wnt activates signaling pathways that are crucial for stem cell regulation, embryonic development, cell polarity proliferation and differentiation and tumorigenesis, just to mention the most relevant functions [161]. There is strong evidence that activation of WNT signal plays a relevant role in initiation and progression of human cancers, rendering FZDs important drug targets for the development of new therapeutic approaches in oncology [160,161,162]. Currently, several anti-FZDs antibodies and small molecule inhibitors are being evaluated as anticancer agents for different types of cancer. There are few reports describing the development of imaging agents for noninvasively probing FZDs in vivo, the majority of which are based on nuclear imaging. The most significant application reported to date is the development of FZD_10_-specific radiolabeled antibodies for theranostics of synovial sarcoma (SS).

**Figure 1:**
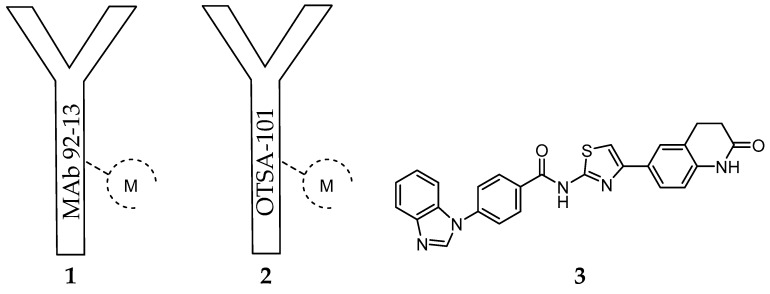
Frizzled receptor (FZD)-targeting molecules. MAb 92-13 (**1**): [^111^In]-**1** (M = ^111^In), [^90^Y]-**1** (M = ^90^Y); OTSA-101 (**2**): [^111^In]-**2** (M = ^111^In), [^90^Y]-**2** (M = ^90^Y).

Considering that FZD_10_ is specifically upregulated at very high level in synovial sarcoma tissues comparing to most normal organs (except for placenta) [163,164], Fukukawa et al. prepared a murine monoclonal antibody (MAb), MAb 92-13 (**1**) [164], with specific binding activity against native FZD_10_ in synovial sarcoma cell lines (Figure 1).

The specificity was confirmed by flow cytometric analysis with fluorescent dyes and radioactive measurement using [^125^I]-**1**. After derivatization of **1** with the bifunctional chelator isothiocyanatobenzyldiethylenetriamine pentaacetic acid (SCN-BZ-DTPA), the resulting protein conjugate was labelled with ^111^In and the biodistribution of the resulting complex [^111^In]-**1** (Figure 1) was assessed in a mice xenograft model of synovial sarcoma (SYO-1-tumor-bearing BALB/c nude mice). The accumulation of radioactivity in the tumor accounted for 11% ID/g after 48 h. Blocking experiments with non-labeled **1** as well as the low accumulation observed in a FZ_D10_ negative SS tumor model confirmed specific tumor accumulation [164]. Moreover, cell studies have also demonstrated that **1** was internalized into the synovial sarcoma cells by a FZD_10_-mediated mechanism. These findings prompted the authors to study the effect of [^90^Y]-**1** (Figure 1) on tumor growth. Interestingly, after a single *i.v.* administration of [^90^Y]-**1** (100 µCi) in the mouse xenograft model, the tumor volume was reduced after treatment, with negligible toxicity associated [164]. Following this first report and aiming to unravel the factors that influence the therapeutic efficacy observed, the authors compared the radiosensitivity, tumor uptake and therapeutic efficacy of **1** in FZD_10_-overexpressing synovial sarcoma cells (SYO-1) with a newly established FZD_10_-transfected DLD-1 cell model (DLD-1/FZD10) [165]. The expression levels of FZD_10_ on the latter cell model (350,000 molecules/cell) is significantly higher than on SYO-1 cells (8000 molecules/cell), as determined by Scatchard plot analysis. As expected from this very different expression level, the biodistribution studies after *i.v.* administration of [^111^In]-**1** have shown higher accumulation of radioactivity in the tumor of DLD-1/FZD10 tumor-bearing mice (49.0 ± 4.2% ID/g) than in SYO-1 tumor-bearing mice (22.0 ± 4.5% ID/g) at 48 h after administration. Surprisingly, the therapeutic efficacy of [^90^Y]-**1** was remarkably higher in SYO-1 tumor-bearing mice than in the DLD-1/FZD10 tumor model, even if higher radiation doses were used during the treatment [165]. Indeed, in the former case, tumor size reduction was observed in all mice treated, while tumor regrowth was not observed in the majority. On the contrary, slow progression was observed in the DLD-1/FZD10 tumor. The authors have assigned this result to the different radiosensitivity of the tumors studied. Brought together, these results validated the use of radiolabeled FZD_10_-specific antibodies for cancer theranostics.

Another important study case of an anti-FZD_10_ therapeutic approach is the humanized chimeric antibody OTSA-101 (**2**) developed by Giraudet et al. (Figure 1). [166]. This anti-FZD_10_ antibody was radiolabeled with ^111^In or ^90^Y after modification with the bifunctional chelator p-SCNBn-CHX-A-DTPA. The resulting radioactive complexes [^111^In]-**2** and [^90^Y]-**2** were evaluated in a first-in-human phase I study in patients with progressive advanced synovial sarcoma following a theranostic approach [166] (Figure 1). Firstly, overall in vivo biodistribution and tumor uptake of [^111^In]-**2** were determined by repeated whole body planar and SPECT-CT scintigraphies. Secondly, the patients with significant tumor uptake were selected for radionuclide therapy with [^90^Y]-**2** [166]. Although no objective response was observed, 3 out of the 8 patients treated achieved a transient stable disease state. Moreover, significant hematotoxicity was observed for most of the patients. Considering these results, the authors envisaged the use of the alternative β-emitter ^177^Lu to radiolabel **2** or a specific anti-FZD_10_ antibody fragment with lower molecular weight, as an approach to surpass the narrow therapeutic index observed [166].

Recently, other research group has evaluated the therapeutic efficacy of the same antibody labelled with an α-particle emitting radionuclide, using a prosthetic group, in the SS xenograft mouse model (SYO-1-tumor-bearing BALB/c nude mice) [167]. In brief, **2** was conjugated to N-succinimidyl-3-(trimethylstannyl)benzoate, and the resulting immunoconjugate was labeled with ^211^At under optimized conditions [168]. A comparative study has demonstrated that the treatment of tumor-bearing mice with [^211^At]-**2** (single doses of 25 and 50 µCi) suppresses the growth of SS xenografts more efficiently than the analogue β-particle emitting antibody [^90^Y]-**2** (50-µCi dose), without relevant toxicity [167]. Remarkably, [^211^At]-**2** (50 µCi) suppressed tumor growth immediately after administration, whereas this effect required several days in the case of [^90^Y]-**2**.

Considering the recent success of radionuclide therapy for the treatment of FZD_10_ overexpressing cancers at preclinical level, namely synovial sarcoma, it is plausible that other types of cancer overexpressing different isoforms of the frizzled receptor (e.g., FZD_7_) would benefit from an analogous approach. Moreover, alternatives to antibodies, such as antibody fragments or small molecules, with relevant receptor-binding properties are still needed. Zhang et al. [169] have proposed novel FZD_7_ inhibitors that act through targeting the receptor’s transmembrane domain (TMD). The identification of low molecular weight FZD_7_ inhibitors was performed by applying structure-based virtual screening targeting the TMD. Homology modeling techniques were employed for the construction of atomic resolution model of the TMDs of FZD_7_ by using its query amino acid sequence and an experimentally available 3D structure of a related homologous protein, which was used as a template. Further, 500,000 structurally diverse compounds were screened through a three-step docking/scoring process using the Glide program. To gain structural insights into FZD_7_ inhibitor binding, a more sophisticated induced-fit docking protocol was performed, allowing binding sites amino acids to be flexible. The results have identified the potent FZD_7_ inhibitor 4-(1H-Benzo[d]imidazol-1-yl)-*N*-(4-(2-oxo-1,2,3,4-tetrahydroquinolin-6-yl)thiazol-2-yl)benzamide (SRI37892, **3**) as a potential chemotherapeutic candidate (Figure 1). Structural-based virtual screening has also been used to predict potential small molecules that binds to the cysteine-rich domain (CRD) of FZD receptors [170,171]. The Glide program was applied for the virtual screening and two small molecule databases were used for structure-based virtual screening (approximately 130,000 compounds) [172]. Five compounds were identified as capable of inhibiting the Wnt signaling, adding important candidates to an effective therapeutic combination.

## 3. Ghrelin Receptor (GHSR-1a)

**Figure 2:**
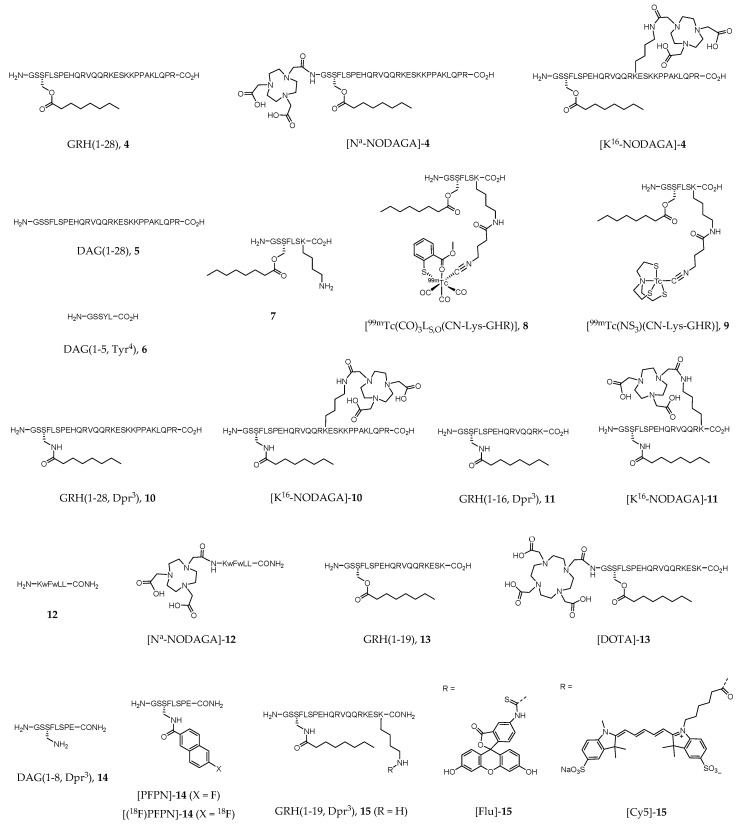
Human ghrelin (GRH(1–28), **4**) and derivatives. Dpr = diaminopropionic acid; w = d-tryptophan.

The growth hormone secretagogue receptor (GHSR), which belongs to the Family A (rodhopsin-like) of seven transmembrane spanning GPCRs [173], consists of two subtypes (1a and 1b), although increasing evidence of novel GHSRs have appeared in the literature in the last decade [174,175,176,177]. The great majority of the scientific work performed focused mainly on the best-known isoform GHSR-1a, also named ghrelin receptor after the discovery of ghrelin as its endogenous ligand (EC_50_ = 1.3 nM, [178]). In healthy individuals, GHSR-1a is widely expressed in CNS, primarily in hypothalamus and pituitary gland, but also in other peripherical organs such as pancreas, gastrointestinal tract, and heart. Human ghrelin, GRH(1–28) (**4**, Figure 2), is a 28 amino acid neuroendocrine peptide hormone N-acylated with an *n*-octanoyl group on the serine side chain at position 3. The unacylated form of ghrelin, human des-acyl-ghrelin (DAG(1–28), **5**, Figure 2), also found in significant levels in human blood, has virtually no affinity to GHSR (EC_50_ > 10,000 nM [179]), and its receptor or function are still unclear. Commonly referenced as a survival system, ghrelin-GHSR axis regulates several metabolic functions including appetite stimulation, gastric motility, suppression of insulin secretion, weight gain, neuroprotection, memory enhancing, and modulation of the immune and cardiovascular systems [173,178,180]. The overexpression of GHSR in some types of cancer (e.g., neuroendocrine tumors of prostate and gonads, among others), or in cardiovascular disease, compared to healthy tissues turned this receptor into a promising drug target for novel therapeutic approaches [181,182]. Thus, a rising number of drug candidates targeting the ghrelin receptor has been proposed, and even some of them successfully achieved phase III clinical trials (e.g., anamorelin, macimorelin and ulimorelin). Indeed, agents targeting GHSR that reached clinical trials account nowadays to 5% of the anti-GPCR candidates, corresponding to the 6th leading subgroup [29]. Consequently, in vivo imaging of GHSR expression also became a subject of rising interest in clinical imaging as well as in drug research. The use of noninvasive imaging techniques would provide better knowledge on biodistribution, biokinetics, metabolic stability, and mode of action of ghrelin receptor ligands in vivo.

^125^I-labeled analogues of GRH(1–28) (**4**) have had a great importance in the assessment of GHSR distribution in human and rat brain [183,184,185]. However, radioiodination of **4** derivatives with ^123^I for SPECT imaging has not been described in the literature in the past years. Interestingly, Wojciuk et al. recently reported the two radioidinated ghrelin derivatives [^131^I]-**5** and [^131^I]-**6** (Figure 2) [186].

Although technetium-99m (^99m^Tc) still accounts for the majority of the imaging procedures in nuclear medicine, there are not many reports on the development of GHSR-specific radioprobes based on that radiometal. One of the few recent reports describes the synthesis and biological evaluation of metal tricarbonyl “2 + 1” type complexes of general formula [M(CO)_3_L_N,O_(CN-Lys-GHR)]^+^ or [M(CO)_3_L_S,O_(CN-Lys-GHR)], in which M = ^99m^Tc or Re; L_N,O_ and L_S,O_ represents *N*,*O*- and *S*,*O*-donor heteroaromatic bidentate ligands, respectively; and CN-Lys-GHR corresponds to a isocyanide monodentate ligand conjugated to a ghrelin derivative (**7**) via the lysine residue through a bifunctional coupling arm [187]. The same authors additionally reported the study of metal(III) tricarbonyl “4 + 1” type complexes of general formula M(NS_3_)(CN-Lys-GHR) (M = ^99m^Tc or Re), where NS_3_ is a tetradentate tripodal trimercaptoamine chelator [187]. Complexes [^99m^Tc(CO)_3_L_S,O_(CN-Lys-GHR)] (**8**, Figure 2) and [^99m^Tc(NS_3_)(CN-Lys-GHR)] (**9**, Figure 2) emerged as the most stable in several aqueous solutions (including human serum) and showed in vitro binding affinity towards GHSR-1a with IC_50_ values in the range 45–54 nM (DU-145 cell model).

Ghrelin derivatives have also been explored for the development of GHSR-specific radioactive probes for PET imaging, such as the work of Chollet et al., who introduced a family of gallium-68 (^68^Ga)-labeled ghrelin agonist and inverse agonist peptides. Biological activity, pharmacokinetic profile and metabolic stability were determined by in vitro and in vivo studies [188]. The agonist compounds [N^α^-NODAGA]-**4**, [K^16^-NODAGA]-**4**, [K^16^-NODAGA]-**10**, [K^16^-NODAGA]-**11**, and inverse agonist [N^α^-NODAGA]-**12** (Figure 2) were prepared by conjugation of the bifunctional chelator 1,4,7-triazacyclononane, 1-glutaric acid-4,7-acetic acid (NODAGA) to GRH(1–28) (**4**) or to ghrelin derivatives **10**–**12**. The corresponding ^nat^Ga complexes were prepared by chelation, purified and fully characterized by the usual analytical techniques in chemistry [188]. Radiolabeled compounds [K^16^-NODAGA(^68^Ga)]-**4**, [K^16^-NODAGA(^68^Ga)]-**10**, [K^16^-NODAGA(^68^Ga)]-**11** and [N^α^-NODAGA(^68^Ga)]-**12** were obtained after reaction of [^68^Ga(OAc)_3_] with the respective precursor peptide conjugates and proceeded to in vivo studies. [K^16^-NODAGA(^68^Ga)]-**4**, NODAGA(^68^Ga)]-**10** and [K^16^-NODAGA(^68^Ga)]-**11** were selected because of their high potency towards GHSR-1a determined in vitro by inositol phosphate turnover assay (EC_50_ = 0.72, 1.91 and 1.41 nM, respectively), whereas [N^α^-NODAGA(^68^Ga)]-**12** was selected since no pharmacokinetic data of ghrelin inverse agonists was known to date [188]. Despite the high affinity to the receptor shown by the ghrelin derived agonists, ex vivo pharmacokinetics and PET imaging studies in Wistar rats demonstrated fast clearance and poor stability of these conjugates in the blood. Thus, the authors concluded that ghrelin motif is not suitable to developing PET imaging agents or to be faced as an appropriate lead for drug development. On the contrary, the inverse agonist [N^α^-NODAGA(^68^Ga)]-**12** exhibited very high stability in blood, large diffusion in tissues, reasonable kidney and liver metabolism without accumulation, and slow blood clearance. However, unspecific diffusion limits its use as a radiotracer for understanding ghrelin receptor signaling [188].

There is also a short report on the use of ^68^Ga-labeled ghrelin derivatives for targeting GHSR-1a associated to prostate cancer cells. Carlie et al. prepared a peptide conjugate of the bifunctional chelator 1,4,7,10-Tetraazacyclododecane-1,4,7,10-tetraacetic acid (DOTA) with a ghrelin(1–19) analogue ([DOTA]-**13**, Figure 2) [189]. The binding affinity found for the metalated complex [DOTA(^nat^Ga)]-**13** (IC_50_ = 9.1 nM) was of the same order of magnitude as for native ghrelin (IC_50_ = 8.1 nM). The radiometalated analogue [DOTA(^68^Ga)]-**13** was synthesized for further biological studies. In vitro competition cell uptake assay using HEK293/GHSR-1a cell model was performed, as well as MicroPET studies in NOD/SCID mice bearing LNCaP tumors. The results showed specific accumulation of the radioactive probe in the tumors, although washout was observed after one hour suggesting low in vivo stability [189].

Aiming to improve the in vivo stability and affinity of ghrelin and ghrelin derivatives towards GHSR-1a, Charron et al. performed a structure-activity study of ghrelin(1–8) in which they identified an analogue containing a fluorine-bearing aromatic prosthetic group (6-fluoro-2-naphthoic acid, PFPN), [PFPN]-**14** (Figure 2) with subnanomolar binding affinity (IC_50_ = 0.11 nM) [190]. A molecular docking protocol using the AUTODOCK 4.2 program was applied to study the interaction between [PFPN]-**14** and the ghrelin receptor [191]. The docking results agree with previous reported results, enhancing the importance of Glu124 at the N-terminal end as an anchor point for binding and its key role in the receptor activation [192]. It is interesting to note that the prosthetic group in position 3 (the same position as of the *n*-octanoyl group of natural ghrelin) is of key importance for recognition and interaction with the receptor. The radiofluorinated analogue 6-[^18^F]-fluoro-2-naphthoic acid-**14** ([(^18^F)-PFPN]-**14**, Figure 2) was also prepared in an overall 3.1% radiochemical yield, however, as far as the authors are aware, no biological evaluation of this probe has been reported yet.

A few reports have also appeared in the literature describing the use of fluorescent ghrelin analogues for live cell imaging of GHSR-expressing cells and organ tissues [193,194,195]. One of the main goals is investigating mechanisms of receptor trafficking or pharmacological agents that target specifically this receptor. Following previous work in the design and synthesis of fluorine- and rhenium(I)-containing ghrelin analogs for GHSR-specific in vivo imaging [196], McGirr et al. synthesized and characterized a new 18 amino acid analog of ghrelin (GRH(1–19, Dpr^3^), **15**) labelled with a fluorescein (Flu) optical dye via an extra Lys^19^ at its C-terminus (compound [Flu]-**15**, Figure 2) [193]. [Flu]-**15** is an agonist of GHSR-1a with in vitro binding affinity similar to that of the endogenous ghrelin (IC_50_ = 9.5 nM). Live cell imaging in CHO/GHSR-1a cells demonstrated the role of this probe in cell surface receptor labelling and internalization. Moreover, the authors demonstrated that this probe binds specifically to heart tissue in situ, by displacement studies with both ghrelin and the known GHSR-1a ligand hexarelin. Therefore, the authors claimed the usefulness of [Flu]-**15** for the development of a fluorescence-based high-throughput screening assay to discover novel GHSR-1a ligands [193]. Interestingly, Lu et al. also conducted studies with [Flu]-**15,** but aiming to assess ex vivo its specificity for human prostate cancer (PCa) cells over healthy adjacent prostate tissue or benign prostatic disease [194]. The main conclusion drawn is the specificity of [Flu]-**1** for PCa (probe signal intensity was 4.7-fold higher in cancer tissue), which suggests the potential of ghrelin analogs to be used as molecular imaging probes for prostatic neoplasms both in localized and metastatic disease [194].

To surpass the drawbacks hampering the use of fluorescein for in vivo imaging, such as the short depth penetration and high degree of tissue autofluorescence in the fluorescein excitation wavelength, McGirr et al. developed the far-red ghrelin analog probe [Cy5]-**15** (Figure 2) [195]. [Cy5]-**15** binding affinity to GHSR-1a (IC_50_ = 25.8 nM) was within the same order of magnitude of [Flu]-**15** and native human ghrelin. Live cell imaging in HEK293/GHSR-1a cells showed cell surface receptor labelling by [Cy5]-**15** [195]. Moreover, the authors also demonstrated that this probe binds specifically to mature cardiomyocytes in a concentration-dependent way, in mouse heart tissue sections. Thus, [Cy5]-**15** may be a useful tool to map GHSR-1a in developing and diseased cardiac tissues [195].

**Figure 3:**
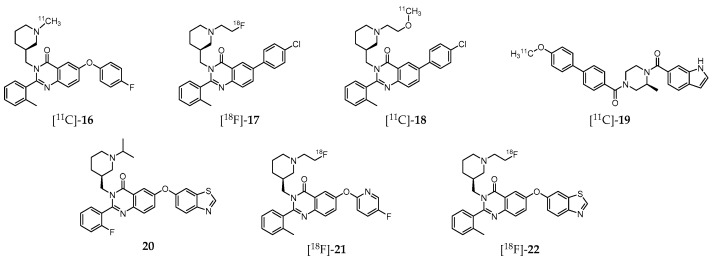
Growth hormone secretagogue receptor (GHSR-1a) specific quinazoline derivatives.

A great deal of effort has been also dedicated to the development of GHSR-specific probes based on quinazoline derivatives for PET imaging (Figure 3). Indeed, aiming to shed light on the mechanism by which ghrelin affects feeding behavior, and thus offering a new therapeutic perspective for the development of effective treatments, Potter et al. introduced the ^11^C-labeled molecule (*S*)-6-(4-fluorophenoxy)-3-((1-[^11^C]methylpiperidin-3-yl)methyl)-2-o-tolylquinazolin-4(3H) -one ([^11^C]-**16**, Figure 3), which displays high GHSR binding affinity (*K*_i_ = 22 nM, CHO-K1 cell model) [197]. Biodistribution studies in CD1 mice have shown that this probe accumulates specifically in the hypothalamus, a region with elevated GHSR density compared to other brain regions with lower GHSR expression. Nevertheless, the cerebral distribution suggests that the radioligand imaging properties may not be enough for further brain studies in human subjects. Moreover, specificity of [^11^C]-**16** to GHSR-1a present on mouse brain compared to GSHR-1a of the peripheral organs was not attained [197].

Further studies by other research group resulted in a series of novel quinazoline-based radioprobes obtained by radiofluorination of 6-(4-chlorophenyl)-3-((1-(2-fluoroethyl)piperidin-3-yl)methyl)-2-(o-tolyl)quinazolin-4(3H)-one ([^18^F]-**17**), by ^11^C-methylation of 6-(4-chlorophenyl)-3-((1-(2-methoxyethyl)piperidin-3-yl)methyl)-2-(o-tolyl)quinazolin-4(3H)-one ([^11^C]-**18**) and by ^11^C-methylation of (*S*)-(4-(1H-indole-6-carbonyl)-3-methylpiperazin-1-yl)(4′-methoxy-[1,1′-biphenyl]-4-yl)methanone ([^11^C]-**19**) (Figure 3) **[198]**. The radiolabeled quinazoline derivatives [^18^F]-**17**, [^11^C]-**18** and [^11^C]-**19** have higher affinity for GHSR-1a (*K*_i_ = 16, 4, and 7 nM, respectively) than [^11^C]-**16** (*K*_i_ = 22 nM) [197]. Surprisingly, ex vivo biodistribution and PET imaging studies in mice showed that brain accumulation of these three tracers was very low. Instead, high levels of [^18^F]-**17** were found in the small intestine, [^11^C]-**18** in the liver, and [^11^C]-**19** in the pancreas. Competition assays by pretreatment with the high potent and selective GSHR-1a inhibitor YIL781 (*K*_i_ = 17 nM), demonstrated that [^11^C]-**19** was an highly selective ligand for GSHR-1a in pancreas, which prompted the authors to claim its usefulness as a PET radiotracer for in vivo imaging of GHSR-1a in that organ [198].

More recently, Hou et al. [199] also conducted experiments on the synthesis and characterization of various quinazolinone derivatives, which resulted in the identification of one fluorine-bearing compound (**20**, Figure 3) with the highest binding affinity for GHSR-1a reported to date (IC_50_ = 20 pM) [199]. Moreover, two lead derivatives with high binding affinities towards GHSR-1a were successfully ^18^F-labeled leading to the radioprobes [^18^F]-**21** (IC_50_ = 20.6 nM) and [^18^F]-**22** (IC_50_ = 9.3 nM; Figure 3). However, these radiofluorinated molecules were not assessed in vivo.

## 4. G protein-Coupled Estrogen Receptor (GPER)

**Figure 4:**
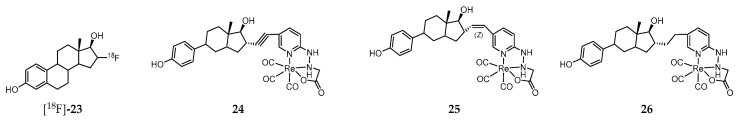
G protein–coupled estrogen receptor (GPER) ligands based on the 17β-estradiol scaffold.

Steroid hormones mediate key physiological functions in the reproductive, cardiovascular, endocrine, nervous, and immune systems. Alteration in those functions may lead to various known pathophysiological conditions [200,201]. The effects of estrogens are exerted through interaction with cellular receptors, namely the nuclear receptor family (ERα and ERβ) and the G protein–coupled receptor family (G protein–coupled estrogen receptor, GPER) [200,201,202]. Whereas ER family regulates gene expression, the GPER family mediates rapid cellular effects through activation of intracellular signaling cascades commonly associated with GPCRs. The clinical relevance of ER imaging in vivo rapidly emerged and, currently, the PET molecular tracer 16α-[^18^F]-fluoro-17β-estradiol ([^18^F]-**23**, Figure 4) plays a unique role in the evaluation and management of breast cancer patients [203,204,205]. However, this radiopharmaceutical does not distinguish the different receptors subtypes.

Considering that assessment of the GPER expression holds potential as a prognostic tool, as a biomarker, and as a therapeutic target in certain cancer types (e.g., ovarian and breast cancer), a strong effort has been pursued towards the development of novel nuclear molecular probes for non-invasive targeting/imaging of that receptor in vivo. One of the main difficulties to overcome within this field is the restrict number of molecules that specifically recognize the GPER with high affinity. Indeed, in most of the GPER-targeted probes developed, binding to “classical” ERs is also observed. That is the case of the neutral Re(I)-tricarbonyl complexes **24**–**26** (Figure 4), stabilized by a tridentate pyridine 2-yl hydrazine chelator and containing a pendant estradiol moiety [206]. These complexes display moderate (**24** and **25**) to low affinity (**26**) for both ERα/β. Interestingly, both complexes **24** and **25** exhibited strong binding to GPER (EC_50_ = 42 and 64 nM, respectively) as assessed by flow cytometry using GPER-transfected COS7 cells that do not express endogenous GPER or ERα/β. Moreover, these complexes were also evaluated in a functional assay based on the rapid receptor-mediated mobilization of intracellular calcium elicited by estrogen ligand binding to ERα/β in transfected COS7 cells expressing ERα or GPER. Alkyne complex **24** led to a rapid increase in intracellular calcium concentrations with both ERα and GPER, whereas the (*Z*)-alkene complex **25** produced lower calcium levels. The biological properties of the radioactive surrogate [^99m^Tc]-**24**, obtained with a radiochemical purity ≥95% after purification and a high specific activity (47.5 TBq of ^99m^Tc/mmol), was assessed in human breast adenocarcinoma MCF-7 cells as well as in virgin female C57BL/6 mice in defined phases of the estrous cycle and in mice bearing MCF-7 and primary human endometrial tumors [207]. The main conclusion of the biodistribution studies in healthy mice was that receptor-mediated uptake was present in all phases of the estrous cycle in reproductive organs and mammary glands but was highest during the diestrous phase of the estrous cycle. As regards the biodistribution studies in tumor-bearing mice, there was significant receptor-mediated accumulation in target tissues and estrogen receptor–expressing tumors (0.67% for MCF-7 tumors and 0.77% for endometrial tumors). However, no discussion is provided regarding the component of the accumulation mediated specifically by the GPER.

**Figure 5:**
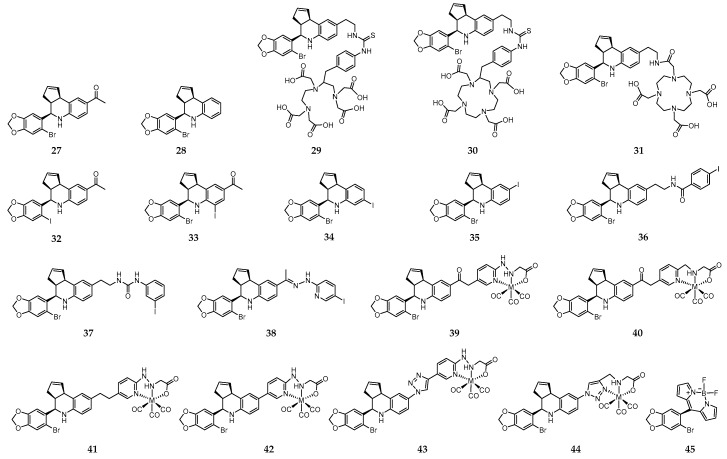
GPER-targeting molecules containing a tetrahydro-3*H*-cyclopenta[c]quinoline core and a Bodipy-derived fluorescent probe. M = Re, ^99m^Tc.

On the quest for synthetic non-steroidal GPER-selective ligands, 1-[4-(6-bromo-benzo[1,3]dioxol-5-yl)-3a,4,5,9b-tetrahydro-3*H*-cyclopenta[c]quinolin-8-yl]-ethanone (**27**) and 4-(6-bromo-benzo[1,3]dioxol-5-yl)-3a,4,5,9b-tetrahydro-3*H*-cyclopenta[c] quinoline (**28**) emerged as high affinity agonist and antagonist, respectively (Figure 5) [208,209].

These molecules share the tetrahydro-3*H*-cyclopenta[c]quinoline scaffold that has been used as a basic motif for the design of novel imaging probes to target GPER. Thus, with the aim of developing radioactive probes to understanding the influence of charge on cell binding, cellular permeability and in vivo tumor imaging, Nayak et al. introduced a family of conjugates combining the tetrahydro-3*H*-cyclopenta[c]quinoline amine moiety with the acyclic bifunctional chelator diethylene triamine pentaacetic acid (DTPA) (**29**, Figure 5) or the cyclic bifunctional chelator 1,4,7,10-tetraazacyclododecane-*N*,*N*′,*N*″,*N*‴-tetraacetic acid (DOTA) (**30** and **31**, Figure 5) [210]. The radioactive complexes [^111^In]-**29**–[^111^In]-**31** were prepared in high radiochemical yield and purity. The corresponding “cold” surrogates [^113^In]-**29**–[^113^In]-**31** were also prepared and fully characterized. In vitro functional assays revealed an effect of charge, as only the neutral analogue [^113^In]-**31** activated GPER-mediated rapid signaling pathways. The binding affinity of this complex to GPER (IC_50_ = 33.9 nM) was comparable to that of the similar small precursor molecules **27** (IC_50_ ≈ 11 nM) and **28** (IC_50_ ≈ 20 nM). Moreover, in parallel assays, no detectable binding of any of the three ^113^In-complexes to ERα was observed, which confirms their high selectivity towards GPER. [^111^In]-**31** was further evaluated in Hec50 endometrial tumor-bearing mice, in which receptor-mediated uptake of the radiotracer in target organs and tumors was observed. However, rapid clearance from the tumor was also observed. Thus, further structural changes were needed towards the design of probes with improved GPER-targeting ability [210].

With that objective in mind, a large family of GPER-selective iodo-substituted quinoline derivatives (**32**–**38**, Figure 5) was also synthesized, characterized and biologically evaluated by Ramesh et al. [211]. These molecules were evaluated against a panel of functional and competitive ligand binding assays using GPER and ERα/β transfected COS7, as well as Hec50 and SKBr3 cells to determine receptor selectivity and potential cross-reactivity. They showed an antagonist effect on GPER and blocking of estrogen-induced PI3K activation and calcium mobilization. Some of them exhibited IC_50_ values lower than 20 nM in competitive binding studies with GPER-expressing human endometrial cancer cells (IC_50_ = 8.4 nM for **37**; IC_50_ = 1.7 nM for **38**). The biodistribution profile of the radioiodinated analogues [^125^I]-**37** and [^125^I]**-38** was assessed in female ovariectomized athymic (NCr) nu/nu mice bearing GPER-expressing human endometrial Hec50 tumors. Although there was receptor-mediated uptake in tumor, adrenal and reproductive organs, neither of the compounds were effective for tumor imaging due to high background, and rapid metabolism. Nevertheless, the authors believed that these compounds constitute a good starting point towards the design of effective imaging probes for GPER with improved targeting properties.

With the goal of overcoming the shortcomings associated to the ^125^I- and ^111^In-containing probes discussed above, Arterburn and coworkers proposed a new family of neutral M(I)-tricarbonyl complexes (M = Re, ^99m^Tc) bearing a pendant tetrahydro-3*H*-cyclopenta[c]quinolone scaffold conjugated through linkers of different nature and length to pyridin-2-yl hydrazine and picolylamine bifunctional chelators (**39**–**44**, Figure 5) [212]. Cell-based assays have shown that two of the rhenium-tricarbonyl complexes, [Re]-**39** and [Re]-**40**, selectively activated the GPER-mediated signaling pathways, similarly to **27**. Interestingly, complex [Re]-**43** with a triazole unit behaves has an antagonist, blocking GPER signaling. The analogue ^99m^Tc-complexes, prepared with high radiochemical yields and purity, presented high stability in biological relevant media. The in vivo GPER-targeting properties of the radioactive complexes [^99m^Tc]-**39**–[^99m^Tc]-**41** were assessed in mice bearing human endometrial and breast cancer cell xenografts. Blocking studies using the GPER-selective agonists estrogen and **27** revealed GPER-specific uptake in adrenals, uterus, and mammary tissue, as well in tumor (0.4–1.1% ID/g) [213]. Brought together, the results established the grounds for the development of novel GPER-specific tumor imaging agents to deepen the knowledge on the role of GPER in estrogen-mediated carcinogenesis and as a target for diagnostic/therapeutic/image-guided drug delivery.

Interestingly, Papalia et al. have developed the GPER-specific fluorescent probe **45** (Figure 5), derived from Bodipy and **27**, for bioimaging purposes and functional dissecting studies. The main conclusion drawn is that **45** is a selective GPER imaging agent that permits visualization of the receptor localization and elucidation of key elements involved in GPER trafficking and the interaction pathways [214].

## 5. Sphingosine-1-Phosphate Receptor 1 (S1PR)

**Figure 6:**
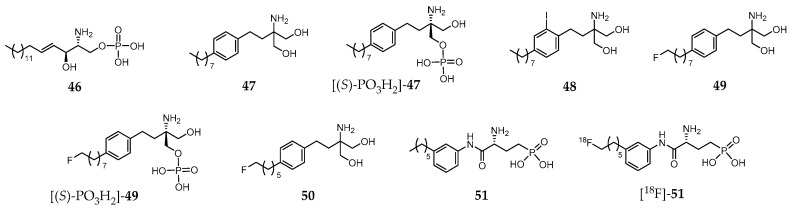
Sphingosine 1-phosphate (S1PR) (**46**) and derivatives.

Sphingosine 1-phosphate (S1P, compound **46**, Figure 6) is a biologically active lysophosphospholipid, which apart from being a cell membrane lipid derivative, acts also as an extracellular signaling molecule.

Its effects are mediated through five specific G protein coupled receptors, S1P receptor 1 (S1PR_1_) to S1P receptor 5 (S1PR_5_). The S1PR_1_–S1PR_3_ are expressed throughout the body, whereas S1PR_4_ is mostly found on immune cells, and S1PR_5_ in lymphocytes, natural killer cells, and oligodendrocytes. The biological functions of **46** include regulation of cellular proliferation, survival, migration, invasion, differentiation and cellular architecture, as well as the control of immune cell trafficking, angiogenesis and vascular integrity. Moreover, it is also implicated in several pathophysiological processes, which include atherosclerosis, respiratory distress, diabetes, inflammation, autoimmune disorders (e.g., multiple sclerosis) and cancer. Owing to the broad range of pathophysiological roles identified, S1PR became a relevant drug target in the last few years and a strong effort has been developed towards finding high affinity and selective ligands. For the sake of example, Fingolimod (**47**, Figure 6) is an approved drug indicated in the treatment of relapsing remitting multiple sclerosis [215]. It is phosphorylated in vivo by sphingosine kinase 2, yielding the biologically active metabolite [(*S*)-PO_3_H_2_]-**47** (Figure 6), a potent S1P receptor agonist. However, this metabolite is a nonselective ligand, which binds to each of the S1PR subtypes except to S1PR_2_ [216].

Within this framework, noninvasive in vitro imaging of S1PR has become a key research topic, as it could potentially contribute to the better understanding of the receptor role in the pathologies where it is involved. Moreover, receptor-specific tracers may serve as a useful tool for drug evaluation and target engagement of S1P receptors modulation strategy.

To date, no S1P receptor imaging agent suitable for clinical studies has been reported. The first attempts to develop an S1PR-targeted imaging probe comprised the preparation and biological evaluation of iodinated analogues of **47**. One of those molecules, **48** (Figure 6), emerged as the most promising as it presents similar properties both to the parent compound and its metabolite. In fact, compound **48** mimics **47** and [(*S*)-PO_3_H_2_]-**47** by displaying similar physicochemical properties, affinity and selectivity for S1P receptors, pharmacokinetic and organ distribution profile, phosphorylation kinetics and phosphate biological activity [217]. Thus, the radioiodinated analogue [^123^I]-**48** was proposed for imaging studies [217].

Aiming to develop ^18^F-based PET imaging probes for targeting S1PR in vivo, Shaikh et al. introduced various fluorinated derivatives of **47**. Among them, the ω-fluorinated analogue **49** and its phosphorylated form [(*S*)-PO_3_H_2_]-**49** (Figure 6) showed remarkable biological activity in vivo [218]. They were able to induce peripheral blood lymphopenia as a characteristic of successful downregulation of the S1P1 and, in the case of [(*S*)-PO_3_H_2_]-**49**, a robust time-dependent phosphorylation of MAPK was observed, indicating its ability to interact with and activate the S1PR_1_ and S1PR_3_ receptors [218]. Surprisingly, another shorter aliphatic chain fluorinated analogue, **50** (Figure 6), presents impaired activity in vivo. The biodistribution of the ^18^F-labeled surrogates [^18^F]-**49** and [^18^F]-**50**, obtained with very high radiochemical purity (>99%), was assessed in C57BL/6 wild type mice. The micro-PET/CT imaging studies showed positive uptake in S1PR-rich tissues, with a slightly increased uptake in the lungs (mainly S1PR_1_) and myocardium (mainly S1PR_3_), as well as in the lymphocyte-containing spleen (mainly S1PR_4_) in the healthy mouse [218].

Following this pioneering work based on an S1PR agonist molecule (**47**), other authors also proposed fluorinated analogues of the S1PR antagonist W146 (**51**, Figure 6). [219]. Those analogues presented in vitro potency (inhibition of the MAPK kinase pathway) comparable to the parent compound. One of them was radiofluorinated, yielding the potential PET probe [^18^F]-**51** (Figure 6) that was later evaluated in adult C57/Bl6 mice (biodistribution studies) [219]. However, defluorination was observed with consequent high accumulation of radioactivity in the bones. These results precluded further evaluation of [^18^F]-**51** as an S1PR PET imaging agent, impelling the authors to suggest metabolically more stable tracers or radiotracers based on different lead structures to overcome this problem [219].

**Figure 7:**
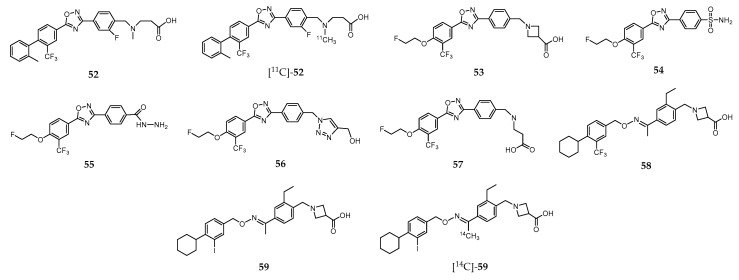
S1PR ligands based on the trifluoromethyl phenyl-oxadiazole scaffold.

Following the successful clinical application of **47** for treating patients with relapsing-remitting forms of multiple sclerosis, considerable research efforts towards the discovery of novel, more efficient, S1PR ligands based on alternative scaffolds have been pursued. Among the great variety of molecules explored, TZ3321 (**52**, Figure 7), a trifluoromethylphenyl-oxadiazol derived compound reported by Merck Serono [220], displays high binding potency for S1PR_1_ (IC_50_ = 2.13 nM), and high selectivity (IC_50_ > 1000 nM) for S1PR_1_ over S1PR_2_ and S1PR_3_.

The radioactive analogue [^11^C]-**52** (Figure 7) was prepared with high radiochemical yield (50–70%, decay corrected), high radiochemical purity (>98%), and high specific activity (2 Ci/μmol at the end of synthesis) [220]. The biodistribution of this PET probe was evaluated in healthy mice (adult male wild-type C57BL/6 mice) and in an animal model for restenosis, namely unilateral femoral artery endothelial denudation (wire-injury) in ApoE-deficient C57BL/6 mice (ApoE^−/−^) maintained on high-fat diet [220]. In both models, biodistribution studies revealed prolonged retention in S1PR_1_-enriched tissues. MicroPET imaging showed higher uptake in the wire-injured arteries of ApoE^−/−^ mice than in injured arteries of wild-type mice. This result was also confirmed by Post-PET autoradiography. Subsequent immunohistochemistry (IHC) staining confirmed higher expression of S1PR_1_ in the neointima of the injured artery of ApoE2/2 mice than in wild-type mice. Brought together, the results allowed the authors to claim that [^11^C]-**52** would be potentially useful for quantification of the S1PR_1_ expression as a biomarker of neointimal hyperplasia [220].

The same research team studied the use of [^11^C]-**52** for the evaluation of S1PR_1_ expression in inflammatory lesions in an experimental autoimmune encephalomyelitis (EAE) rat model of multiple sclerosis (MS) [221]. The main conclusion was that EAE-induced upregulation of S1PR_1_ expression in rat lumbar spinal cord could be assessed by PET imaging, highlighting its potential use to detect neuroinflammatory response in patients with MS and other CNS diseases [221]. Moreover, the efficacy of [^11^C]-**52** to assessing the change of S1PR_1_ levels in the arterial wall during acute vascular inflammation by PET imaging was also studied [222]. Micro-PET/CT imaging after intravenous injection of [^11^C]-**52** allowed to visualize the increased expression of S1PR_1_ in infiltrated inflammatory cells in carotid artery 72 h after balloon over inflation injury in male adult Sprague-Dawley rats. This selective accumulation was confirmed by ex vivo autoradiography and immunohistological analysis. The results pointed to the conclusion that [^11^C]-**52** could potentially be used as a PET radiotracer for monitoring early inflammatory response and the efficacy of antivascular inflammation therapy [222].

More recently, Liu et al. demonstrated that microPET/CT imaging with [^11^C]-**52** allowed to specifically identify upregulated S1PR1 expression in mouse atherosclerotic plaques, confirmed by immunostaining methods in aortic plaques in the murine model of atherosclerosis as well as in human femoral atherosclerotic plaques [223]. These results paved the way towards the development of novel imaging probes for the noninvasive detection and visualization of atherosclerotic plaques.

**Figure 8:**
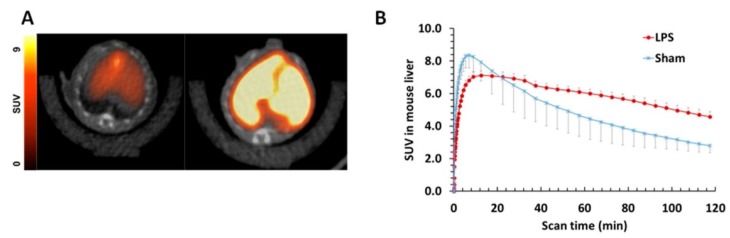
(**A**) Representative summed 120 min transverse PET/CT images of [^18^F]-53 in a sham (left) and LPS-treated (right) mouse. (**B**) Liver time−activity curves (TAC) of [^18^F]53 standardized uptake values (SUV) in sham and LPS-treated mice. Adapted with permission from A. J. Rosenberg et al. J. Med. Chem. 2016, 59 (13), 6201–6220 [216]. Copyright © 2016 American Chemical Society.

Also with the purpose of finding potent and selective PET imaging tracers for S1PR_1_, Rosenberg et al. synthesized and characterized two families of fluorinated compounds containing a benzoxazole core or an oxadiazole core [216]. Their potency to S1PR_1_, S1PR_2_, and S1PR_3_ was determined by competitive inhibition assays with radiolabeled [^32^P]-**46**. One of the compounds of the oxadiazole family, **53** (Figure 7), emerged as the most promising due to its unprecedent potency (IC_50_ = 2.6 nM for S1PR_1_) and remarkable selectivity (>100-fold for S1PR_1_ versus S1PR_2_/S1PR_3_). The radiofluorinated analogue [^18^F]-**53** was obtained in ca. 26% radiochemical yield, with 98% chemical and radiochemical purity, and high specific activity. The biodistribution of [^18^F]-**53** was evaluated in adult male Sprague−Dawley (SD) rats and in a mouse model of liver inflammation (lipopolysaccharide(LPS)-treated mice). Immunohistochemical analysis staining studies confirmed that S1PR_1_ expression was increased in the liver of LPS-treated mice. For the healthy SD rats, accumulation in organs with high S1PR_1_ expression (e.g., heart and pancreas) was observed without evidence of defluorination; whereas for the LPS-treated mice, microPET imaging studies have shown high accumulation of radioactivity in the liver (Figure 8A, right) as compared to non-treated animals (Figure 8A, left).

**Figure 9:**
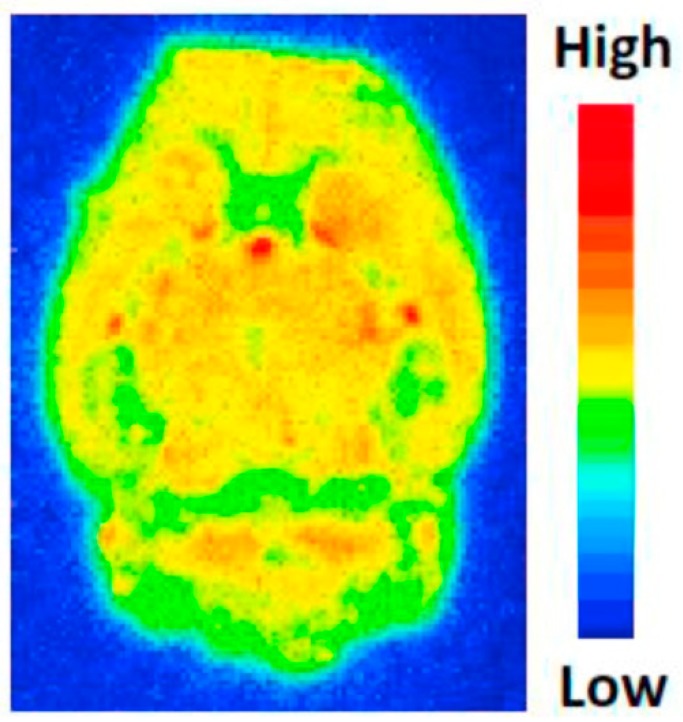
Ex vivo autoradiography of [^18^F]-56 in normal rat brain. “Figure 3. Ex vivo autoradiography of [^18^F]12b in rat brain” from the following article: Luo Z. et al. Eur. J. Med. Chem. 2018, 150, 796–808. doi: 10.1016/j.ejmech.2018.03.035. Syntheses and in vitro evaluation of new S1PR1 compounds and initial evaluation of a lead F-18 radiotracer in rodents. [224]. Copyright © Elsevier.

Additionally, in vitro autoradiography showed increased binding in the liver of LPS-treated mice compared to controls. Interestingly, parallel microPET imaging experiments with radioactive probes for assessing liver function, namely [^15^O]-H_2_O and [^99m^Tc]-mebrofenin to evaluating blood flow and hepatobiliary clearance, respectively, strongly suggested that uptake of [^18^F]-**53** was related with S1PR_1_ expression. Based on these results, the authors claimed that [^18^F]-**53** could be considered an S1PR_1_-specific PET tracer with high potential for in vivo imaging of this target, and allowing the quantitation of receptor expression in response to inflammation [216]. However, [^18^F]-**53** was unable to penetrate the blood–brain barrier (BBB), which would preclude its use as a radiotracer for visualization of neuroinflammation in vivo. Thus, aiming to overcome this issue, Tu and coworkers developed a new family of ligands in which the azetidine-3-carboxylic acid moiety in **53** was replaced by various hydrophilic groups [224]. Among the various molecules evaluated by in vitro binding assays, compounds **54**–**57** (Figure 7) presented the highest S1PR binding affinities, with IC_50_ values ranging from 6.3 to 15 nM. These molecules also showed high selectivity for S1PR_1_ versus other S1P receptor subtypes (IC_50_ > 1000 nM for S1PR_2_–S1PR_5_). The radiolabeled surrogate [^18^F]-**56** was obtained in ca. 14.1% radiochemical yield and high radiochemical purity (>98%) [224]. Biodistribution and ex vivo autoradiography studies of this radioprobe in rodents demonstrated good specific activity, stability and remarkable ability to cross the BBB with good brain retention as shown in Figure 9.

Further studies, namely in vitro autoradiography of brain slices of LPS-induced neuroinflammation mice model demonstrated that specific ligands of S1PR_1_ reduce the uptake of [^18^F]-**56**, suggesting that it has specific binding towards S1PR_1_. Thus, [^18^F]-**56** has the potential to be an efficient PET imaging tracer for visualization of S1PR_1_ expression in the brain [224].

Siponimod (**58**, Figure 7) is a selective agonist of S1PR_1_ and SP1R_5_ that is currently in advanced Phase III clinical trials across 292 hospital clinics in 31 countries for treating secondary progressive multiple sclerosis (SPMS) [225]. To further investigating the therapeutic efficacy of **58**, namely the assessement of its tissue distribution in patients and, more important, its ability to cross the BBB and to accumulate at the sites of MS lesions, Briard et al. developed an iodinated analogue, **59** (Figure 7), with the potential to be labeled with ^123^I for SPECT imaging [226]. This analogue showed to be very similar to **58**, including its affinity and selectivity to S1P receptors, overall physicochemical properties, blood pharmacokinetics and tissue distribution (determined by whole-body autoradiography using [^14^C]-**59**, Figure 7). Molecule **59** was further radiolabeled with ^123^I and preliminary SPECT imaging studies in nonhuman primates confirmed [^123^I]-**59** as a promising SPECT imaging agent to investigate the distribution of **58** in the human central nervous system [227].

## 6. Concluding Remarks and Perspectives

We have surveyed recent efforts towards the design and introduction of innovative molecular imaging and/or theranostic agents targeting the FZD, GPER, GHSR-1a, and S1PR, highlighting the clinical relevance of such efforts. Among these GPCRs, the GHSR-1a is undoubtedly the one that has been most exploited, and various radiolabeled tracers, namely those derived from human ghrelin, were developed for noninvasive imaging of GHSR-1a at the preclinical level. However, to the best of our knowledge, no imaging tracer has reached clinical trials yet. Nevertheless, considering that the number of patents covering this topic is continuously increasing, also a rising importance of GHSR-1a in molecular imaging and precision medicine is predicted [228,229]. Contrary to the GHSR-1a, the number of molecules developed to targeting the FZD, mainly antibodies, is still scarce and exciting achievements in the development of FZD-targeted drugs and effective imaging agents for assessing noninvasively the in vivo expression of FZD receptors are expected in the near future. As regards the GPER, a great deal of effort has been directed towards the design of highly specific GPER-ligands and imaging agents based mainly on derivatives containing the tetrahydro-3H-cyclopenta[c]quinolone scaffold. However, the number of selective GPER-targeting molecules is still limited, and exploitation of structural diversity could lead to a higher number of efficient compounds, including peptides or engineered antibodies. Concerning the S1PR, also the type of molecules used to image this receptor in vivo by nuclear imaging is quite limited, as only sphingosine 1-phosphate derivatives and trifluoromethyl phenyl-oxadiazole-containing compounds have been described. Therefore, the introduction of novel S1PR-targeting molecules is decisive for the development of effective molecular imaging agents to probe that GPCR in vivo.

As an overall conclusion, we are of the opinion that further successful achievements in molecular imaging of the selected GPCRs, and consequently in theranostic approaches, are still much dependent on the availability of novel molecules with high selectivity and affinity. Integrated methodologies combining in-silico and experimental approaches could decisively contribute to that goal and should be further explored. Moreover, we are also of the opinion that molecular imaging of those targets will have a huge impact in drug discovery and address relevant unmet needs in medicine.

## References

[1] Collins D.C., Sundar R., Lim J.S.J., Yap T.A. (2017). Towards Precision Medicine in the Clinic: From Biomarker Discovery to Novel Therapeutics. Trends Pharmacol. Sci..

[2] Workman P., de Bono J. (2008). Targeted therapeutics for cancer treatment: Major progress towards personalised molecular medicine. Curr. Opin. Pharmacol..

[3] Moscow J.A., Fojo T., Schilsky R.L. (2018). The Evidence Framework for Precision Cancer Medicine. Nat. Rev. Clin. Oncol..

[4] Jameson J.L., Longo D.L. (2015). Precision Medicine—Personalized, Problematic, and Promising. N. Engl. J. Med..

[5] Dugger S.A., Platt A., Goldstein D.B. (2018). Drug development in the era of precision medicine. Nat. Rev. Drug Discov..

[6] Jadvar H. (2017). Targeted Radionuclide Therapy: An Evolution Toward Precision Cancer Treatment. Am. J. Roentgenol..

[7] Giardino A., Gupta S., Olson E., Sepulveda K., Lenchik L., Ivanidze J., Rakow-Penner R., Patel M.J., Subramaniam R.M., Ganeshan D. (2017). Role of Imaging in the Era of Precision Medicine. Acad. Radiol..

[8] Herold C.J., Lewin J.S., Wibmer A.G., Thrall J.H., Krestin G.P., Dixon A.K., Schoenberg S.O., Geckle R.J., Muellner A., Hricak H. (2016). Imaging in the Age of Precision Medicine: Summary of the Proceedings of the 10th Biannual Symposium of the International Society for Strategic Studies in Radiology. Radiology.

[9] Ghasemi M., Nabipour I., Omrani A., Alipour Z., Assadi M. (2016). Precision medicine and molecular imaging: New targeted approaches toward cancer therapeutic and diagnosis. Am. J. Nucl. Med. Mol. Imaging.

[10] McDermott S., Kilcoyne A. (2016). Molecular imaging-its current role in cancer. QJM Int. J. Med..

[11] Lu Z.R., Minko T. (2017). Molecular imaging for precision medicine. Adv. Drug Deliv. Rev..

[12] Ametamey S.M., Honer M., Schubiger P.A. (2008). Molecular imaging with PET. Chem. Rev..

[13] Wester H.J. (2007). Nuclear imaging probes: From bench to bedside. Clin. Cancer Res..

[14] Sharma S. (2016). PET Radiopharmaceuticals for Personalized Medicine. Curr. Drug Targets.

[15] McMahon M.T., Chan K.W.Y. (2014). Developing MR Probes for Molecular Imaging. Emerg. Appl. Mol. Imaging Oncol..

[16] Sim N., Parker D. (2015). Critical design issues in the targeted molecular imaging of cell surface receptors. Chem. Soc. Rev..

[17] Herschman H.R. (2003). Molecular imaging: Looking at problems, seeing solutions. Science.

[18] Weissleder R., Pittet M.J. (2008). Imaging in the era of molecular oncology. Nature.

[19] Price E., Orvig C. (2014). Matching chelators to radiometals for radiopharmaceuticals. Chem. Soc. Rev..

[20] Yordanova A., Eppard E., Krupig S., Bundschuh R.A., Schonberger S., Gonzalez-Carmona M., Feldmann G., Ahmadzadehfar H., Essler M. (2017). TheranosticsTheranostics in nuclear medicine practice. Oncotargets Ther..

[21] Choudhury P., Gupta M. (2017). Personalized & Precision Medicine in Cancer: A Theranostic Approach. Curr. Radiopharm..

[22] Notni J., Wester H.-J. (2018). Re-thinking the role of radiometal isotopes: Towards a future concept for theranostic radiopharmaceuticals. J. Label. Compd. Radiopharm..

[23] Cutler C.S., Hennkens H.M., Sisay N., Huclier-Markai S., Jurisson S.S. (2013). Radiometals for Combined Imaging and Therapy. Chem. Rev..

[24] Ballinger J.R. (2018). Theranostic radiopharmaceuticals: Established agents in current use. Br. J. Radiol..

[25] Ahmadzadehfar H., Essler M. (2018). It is time to move forward into the era of Theranostics. Ejnmmi Res..

[26] Santos-Cuevas C., Ferro-Flores G., Garcia-Perez F.O., Jimenez-Mancilla N., Ramirez-Nava G., Ocampo-Garcia B., Luna-Gutierrez M., Azorin-Vega E., Davanzo J., Soldevilla-Gallardo I. (2018). Lu-177-DOTA-HYNIC-Lys(Nal)-Urea-Glu: Biokinetics, Dosimetry, and Evaluation in Patients with Advanced Prostate Cancer. Contrast Media Mol. Imaging.

[27] Eberlein U., Cremonesi M., Lassmann M. (2017). Individualized Dosimetry for Theranostics: Necessary, Nice to Have, or Counterproductive?. J. Nucl. Med..

[28] Li T.T., Ao E.C.I., Lambert B., Brans B., Vandenberghe S., Mok G.S.P. (2017). Quantitative Imaging for Targeted Radionuclide Therapy Dosimetry—Technical Review. Theranostics.

[29] Hauser A.S., Attwood M.M., Rask-Andersen M., Schioth H.B., Gloriam D.E. (2017). Trends in GPCR drug discovery: New agents, targets and indications. Nat. Rev. Drug Discov..

[30] Hicks R.J. (2017). Citius, Altius, Fortius: An Olympian Dream for Theranostics. J. Nucl. Med..

[31] Graham M.M., Gu X.M., Ginader T., Breheny P., Sunderland J.J. (2017). Ga-68-DOTATOC Imaging of Neuroendocrine Tumors: A Systematic Review and Metaanalysis. J. Nucl. Med..

[32] Bodei L., Ambrosini V., Herrmann K., Modlin I. (2017). Current Concepts in Ga-68-DOTATATE Imaging of Neuroendocrine Neoplasms: Interpretation, Biodistribution, Dosimetry, and Molecular Strategies. J. Nucl. Med..

[33] Ambrosini V., Morigi J.J., Nanni C., Castellucci P., Fanti S. (2015). Current status of PET imaging of neuroendocrine tumours (18F FDOPA, 68Ga tracers, 11C/18F-HTP). Q. J. Nucl. Med. Mol. Imaging.

[34] Ambrosini V., Campana D., Tomassetti P., Fanti S. (2012). Ga-68-labelled peptides for diagnosis of gastroenteropancreatic NET. Eur. J. Nucl. Med. Mol. Imaging.

[35] Pepe G., Moncayo R., Bombardieri E., Chiti A. (2012). Somatostatin receptor SPECT. Eur. J. Nucl. Med. Mol. Imaging.

[36] Santhanam P., Chandramahanti S., Kroiss A., Yu R., Ruszniewski P., Kumar R., Taieb D. (2015). Nuclear imaging of neuroendocrine tumors with unknown primary: Why, when and how?. Eur. J. Nucl. Med. Mol. Imaging.

[37] Cuccurullo V., Prisco M.R., Di Stasio G.D., Mansi L. (2017). Nuclear Medicine in Patients with NET: Radiolabeled Somatostatin Analogues and their Brothers. Curr. Radiopharm..

[38] Hope T.A., Pampaloni M.H., Flavell R.R., Nakakura E.K., Bergsland E.K. (2017). Somatostatin receptor PET/MRI for the evaluation of neuroendocrine tumors. Clin. Transl. Imaging.

[39] Barrio M., Czernin J., Fanti S., Ambrosini V., Binse I., Du L., Eiber M., Herrmann K., Fendler W.P. (2017). The Impact of Somatostatin Receptor-Directed PET/CT on the Management of Patients with Neuroendocrine Tumor: A Systematic Review and Meta-Analysis. J. Nucl. Med..

[40] Cives M., Strosberg J. (2017). Radionuclide Therapy for Neuroendocrine Tumors. Curr. Oncol. Rep..

[41] Pool S.E., Krenning E.P., Koning G.A., van Eijck C.H.J., Teunissen J.J.M., Kam B., Valkema R., Kwekkeboom D.J., de Jong M. (2010). Preclinical and Clinical Studies of Peptide Receptor Radionuclide Therapy. Semin. Nucl. Med..

[42] van Essen M., Sundin A., Krenning E.P., Kwekkeboom D.J. (2014). Neuroendocrine tumours: The role of imaging for diagnosis and therapy. Nat. Rev. Endocrinol..

[43] Werner R.A., Bluemel C., Allen-Auerbach M.S., Higuchi T., Herrmann K. (2015). (68)Gallium- and (90)Yttrium-/(177)Lutetium: “theranostic twins” for diagnosis and treatment of NETs. Ann. Nucl. Med..

[44] Fendler W.P., Baum R.P. (2017). NTR Is the New SSTR? Perspective for Neurotensin Receptor 1 (NTR)-Directed Theranostics. J. Nucl. Med..

[45] Emrarian I., Sadeghzadeh N., Abedi S.M., Abediankenari S. (2018). New neurotensin analogue radiolabeled by 99m-technetium as a potential agent for tumor identification. Chem. Biol. Drug Des..

[46] Maschauer S., Prante O. (2017). Radiopharmaceuticals for imaging and endoradiotherapy of neurotensin receptor-positive tumors. J. Label. Compd. Radiopharm..

[47] Baum R.P., Singh A., Schuchardt C., Kulkarni H.R., Klette I., Wiessalla S., Osterkamp F., Reineke U., Smerling C. (2017). 177Lu-3BP-227 for neurotensin receptor 1-targeted therapy of metastatic pancreatic adenocarcinoma—First clinical results. J. Nucl. Med. Off. Publ. Soc. Nucl. Med..

[48] Deng H., Wang H., Zhang H., Wang M., Giglio B., Ma X., Jiang G., Yuan H., Wu Z., Li Z. (2017). Imaging Neurotensin Receptor in Prostate Cancer with Cu-64-Labeled Neurotensin Analogs. Mol. Imaging.

[49] Schulz J., Rohracker M., Stiebler M., Goldschmidt J., Stober F., Noriega M., Pethe A., Lukas M., Osterkamp F., Reineke U. (2017). Proof of Therapeutic Efficacy of a Lu-177-Labeled Neurotensin Receptor 1 Antagonist in a Colon Carcinoma Xenograft Model. J. Nucl. Med..

[50] Schulz J., Rohracker M., Stiebler M., Goldschmidt J., Grosser O.S., Osterkamp F., Pethe A., Reineke U., Smerling C., Amthauer H. (2016). Comparative Evaluation of the Biodistribution Profiles of a Series of Nonpeptidic Neurotensin Receptor-1 Antagonists Reveals a Promising Candidate for Theranostic Applications. J. Nucl. Med..

[51] Ferreira C.D., Fuscaldi L.L., Townsend D.M., Rubello D., de Barros A.L.B. (2017). Radiolabeled bombesin derivatives for preclinical oncological imaging. Biomed. Pharmacother..

[52] Mansi R., Minamimoto R., Macke H., Iagaru A.H. (2016). Bombesin-Targeted PET of Prostate Cancer. J. Nucl. Med..

[53] Mansi R., Fleischmann A., Macke H.R., Reubi J.C. (2013). Targeting GRPR in urological cancers -from basic research to clinical application. Nat. Rev. Urol..

[54] Sancho V., Di Florio A., Moody T.W., Jensen R.T. (2011). Bombesin Receptor-Mediated Imaging and Cytotoxicity: Review and Current Status. Curr. Drug Deliv..

[55] Schroeder R.P.J., van Weerden W.M., Bangma C., Krenning E.P., de Jong M. (2009). Peptide receptor imaging of prostate cancer with radiolabelled bombesin analogues. Methods.

[56] Mansour N., Paquette M., Ait-Mohand S., Dumulon-Perreault V., Guerin B. (2018). Evaluation of a novel GRPR antagonist for prostate cancer PET imaging: Cu-64-DOTHA(2)-PEG-RM26. Nucl. Med. Biol..

[57] Ceci F., Castellucci P., Cerci J.J., Fanti S. (2017). New aspects of molecular imaging in prostate cancer. Methods.

[58] Wieser G., Popp I., Rischke H.C., Drendel V., Grosu A.L., Bartholoma M., Weber W.A., Mansi R., Wetterauer U., Schultze-Seemann W. (2017). Diagnosis of recurrent prostate cancer with PET/CT imaging using the gastrin-releasing peptide receptor antagonist Ga-68-RM2: Preliminary results in patients with negative or inconclusive F-18 Fluoroethylcholine-PET/CT. Eur. J. Nucl. Med. Mol. Imaging.

[59] Sonni I., BarattO L., Iagaru A. (2017). Imaging of Prostate Cancer Using Gallium-68-Labeled Bombesin. PET Clin..

[60] Maffioli L., Florimonte L., Costa D.C., Castanheira J.C., Grana C., Luster M., Bodei L., Chinol M. (2015). New radiopharmaceutical agents for the treatment of castration-resistant prostate cancer. Q. J. Nucl. Med. Mol. Imaging.

[61] Moreno P., Ramos-Alvarez I., Moody T.W., Jensen R.T. (2016). Bombesin related peptides/receptors and their promising therapeutic roles in cancer imaging, targeting and treatment. Expert Opin. Ther. Targets.

[62] Maina T., Nock B.A., Kulkarni H., Singh A., Baum R.P. (2017). Theranostic Prospects of Gastrin-Releasing Peptide Receptor-Radioantagonists in Oncology. PET Clin..

[63] Kim K., Zhang H.W., La Rosa S., Jebiwott S., Desai P., Kimm S., Scherz A., O’Donoghue J.A., Weber W.A., Coleman J.A. (2017). Bombesin Antagonist-Based Radiotherapy of Prostate Cancer Combined with WST-11 Vascular Targeted Photodynamic Therapy. Clin. Cancer Res..

[64] Maina T., Nock B.A. (2017). From Bench to Bed New Gastrin-Releasing Peptide Receptor-Directed Radioligands and Their Use in Prostate Cancer. PET Clin..

[65] Reynolds T.S., Bandari R.P., Jiang Z.R., Smith C.J. (2016). Lutetium-177 Labeled Bombesin Peptides for Radionuclide Therapy. Curr. Radiopharm..

[66] Roosenburg S., Laverman P., van Delft F.L., Boerman O.C. (2011). Radiolabeled CCK/gastrin peptides for imaging and therapy of CCK2 receptor-expressing tumors. Amino Acids.

[67] Maina T., Konijnenberg M.W., KolencPeitl P., Garnuszek P., Nock B.A., Kaloudi A., Kroselj M., Zaletel K., Maecke H., Mansi R. (2016). Preclinical pharmacokinetics, biodistribution, radiation dosimetry and toxicity studies required for regulatory approval of a phase I clinical trial with In-111-CP04 in medullary thyroid carcinoma patients. Eur. J. Pharm. Sci..

[68] Trejtnar F., Laznickova A., Laznicek M., Novy Z., Maina T., Nock B.A., Behe M. (2012). Distribution, Elimination, and Renal Handling of (99m)Technetium-Demogastrin 1. Cancer Biother. Radiopharm..

[69] Roy J., Putt K.S., Coppola D., Leon M.E., Khalil F.K., Centeno B.A., Clark N., Stark V.E., Morse D.L., Low P.S. (2016). Assessment of cholecystokinin 2 receptor (CCK2R) in neoplastic tissue. Oncotarget.

[70] Pawlak D., Rangger C., Peitl P.K., Garnuszek P., Maurin M., Ihli L., Kroselj M., Maina T., Maecke H., Erba P. (2016). From preclinical development to clinical application: Kit formulation for radiolabelling the minigastrin analogue CP04 with In-111 for a first-in-human clinical trial. Eur. J. Pharm. Sci..

[71] Kaloudi A., Nock B.A., Lymperis E., Valkema R., Krenning E.P., de Jong M., Maina T. (2016). Impact of clinically tested NEP/ACE inhibitors on tumor uptake of (111)ln-DOTA MG11-first estimates for clinical translation. Ejnmmi Res..

[72] Kaloudi A., Nock B.A., Lymperis E., Krenning E.P., de Jong M., Maina T. (2016). Improving the In Vivo Profile of Minigastrin Radiotracers: A Comparative Study Involving the Neutral Endopeptidase Inhibitor Phosphoramidon. Cancer Biother. Radiopharm..

[73] Kaloudi A., Nock B.A., Lymperis E., Krenning E.P., de Jong M., Maina T. (2016). Tc-99m-labeled gastrins of varying peptide chain length: Distinct impact of NEP/ACE-inhibition on stability and tumor uptake in mice. Nucl. Med. Biol..

[74] Kaloudi A., Nock B.A., Krenning E.P., Maina T., De Jong M. (2015). Radiolabeled gastrin/CCK analogs in tumor diagnosis: Towards higher stability and improved tumor targeting. Q. J. Nucl. Med. Mol. Imaging.

[75] Nock B.A., Maina T., Krenning E.P., de Jong M. (2014). “To Serve and Protect”: Enzyme Inhibitors as Radiopeptide Escorts Promote Tumor Targeting. J. Nucl. Med..

[76] Keller M., Maschauer S., Brennauer A., Tripal P., Koglin N., Dittrich R., Bernhardt G., Kuwert T., Wester H.J., Buschauer A. (2017). Prototypic F-18-Labeled Argininamide-Type Neuropeptide Y Y1R Antagonists as Tracers for PET Imaging of Mammary Carcinoma. ACS Med. Chem. Lett..

[77] Zhang C., Pan J., Lin K.S., Dude I., Lau J., Zeisler J., Merkens H., Jenni S., Guerin B., Benard F. (2016). Targeting the Neuropeptide Y1 Receptor for Cancer Imaging by Positron Emission Tomography Using Novel Truncated Peptides. Mol. Pharm..

[78] Hofmann S., Maschauer S., Kuwert T., Beck-Sickinger A.G., Prante O. (2015). Synthesis and in Vitro and in Vivo Evaluation of an F-18-Labeled Neuropeptide Y Analogue for Imaging of Breast Cancer by PET. Mol. Pharm..

[79] Morgat C., Mishra A.K., Varshney R., Allard M., Fernandez P., Hindie E. (2014). Targeting Neuropeptide Receptors for Cancer Imaging and Therapy: Perspectives with Bombesin, Neurotensin, and Neuropeptide-Y Receptors. J. Nucl. Med..

[80] Winterdahl M., Audrain H., Landau A.M., Smith D.F., Bonaventure P., Shoblock J.R., Carruthers N., Swanson D., Bender D. (2014). PET Brain Imaging of Neuropeptide Y2 Receptors Using N-C-11-Methyl-JNJ-31020028 in Pigs. J. Nucl. Med..

[81] Hostetler E.D., Sanabria-Bohorquez S., Fan H., Zeng Z.Z., Gantert L., Williams M., Miller P., O’Malley S., Kameda M., Ando M. (2011). Synthesis, characterization, and monkey positron emission tomography (PET) studies of F-18 Y1-973, a PET tracer for the neuropeptide Y Y1 receptor. Neuroimage.

[82] Chatenet D., Cescato R., Waser B., Erchegyi J., Rivier J.E., Reubi J.C. (2011). Novel dimeric DOTA-coupled peptidic Y-1-receptor antagonists for targeting of neuropeptide Y receptor-expressing cancers. Ejnmmi Res..

[83] Guerin B., Dumulon-Perreault V., Tremblay M.C., Ait-Mohand S., Fournier P., Dubuc C., Authier S., Benard F. (2010). Lys(DOTA)(4) BVD15, a novel and potent neuropeptide Y analog designed for Y-1 receptor-targeted breast tumor imaging. Bioorg. Med. Chem. Lett..

[84] Khan I.U., Zwanziger D., Bohme I., Javed M., Naseer H., Hyder S.W., Beck-Sickinger A.G. (2010). Breast-Cancer Diagnosis by Neuropeptide Y Analogues: From Synthesis to Clinical Application. Angew. Chem.-Int. Ed..

[85] Smith D., Winterdahl M., Audrain H., Landau A., Bonaventure P., Shoblock J., Carruthers N., Swanson D., Bender D. (2013). PET brain imaging of neuropeptide Y2 receptors. Eur. Neuropsychopharmacol..

[86] Higuchi T., Rischpler C., Fukushima K., Isoda T., Xia J.S., Javadi M.S., Szabo Z., Dannals R.F., Mathews W.B., Bengel F.M. (2013). Targeting of Endothelin Receptors in the Healthy and Infarcted Rat Heart Using the PET Tracer F-18-FBzBMS. J. Nucl. Med..

[87] Chen X.Y., Werner R.A., Javadi M.S., Maya Y., Decker M., Lapa C., Herrmann K., Higuchi T. (2015). Radionuclide Imaging of Neurohormonal System of the Heart. Theranostics.

[88] Michel K., Buther K., Law M.P., Wagner S., Schober O., Hermann S., Schafers M., Riemann B., Holtke C., Kopka K. (2011). Development and Evaluation of Endothelin-A Receptor (Radio)Ligands for Positron Emission Tomography. J. Med. Chem..

[89] Bai M., Bornhop D.J. (2012). Recent Advances in Receptor-Targeted Fluorescent Probes for In Vivo Cancer Imaging. Curr. Med. Chem..

[90] Holtke C., Faust A., Breyholz H.J., Kopka K., Schober O., Riemann B., Bremer C., Schafers M., Wagner S. (2009). Non-Invasive Approaches to Visualize the Endothelin Axis In Vivo Using State-of-the-Art Molecular Imaging Modalities. Mini-Rev. Med. Chem..

[91] Holtke C., Law M.P., Wagner S., Kopka K., Faust A., Breyholz H.J., Schober O., Bremer C., Riemann B., Schafers M. (2009). PET-compatible endothelin receptor radioligands: Synthesis and first in vitro and in vivo studies. Bioorg. Med. Chem..

[92] Mathews W.B., Murugesan N., Xia J., Scheffell U., Hilton J., Ravert H.T., Dannals R.F., Szabo Z. (2008). Synthesis and in vivo evaluation of novel PET radioligands for imaging the endothelin-A receptor. J. Nucl. Med..

[93] Zhang C.C., Lin K.S., Benard F. (2017). Molecular Imaging and Radionuclide Therapy of Melanoma Targeting the Melanocortin 1 Receptor. Mol. Imaging.

[94] Zhang C.C., Zhang Z.X., Lin K.S., Pan J.H., Dude I., Hundal-Jabal N., Colpo N., Benard F. (2017). Preclinical Melanoma Imaging with Ga-68-Labeled alpha-Melanocyte-Stimulating Hormone Derivatives Using PET. Theranostics.

[95] Teixeira V., Fernandez M., Oddone N., Zhang X.L., Gallazzi F., Cerecetto H., Gambini J.P., Porcal W., Cabral P., Quinn T.P. (2017). The Effect of A Hexanoic Acid Linker Insertion on the Pharmacokinetics and Tumor Targeting Properties of the Melanoma Imaging Agent 99mTc-HYNIC-cycMSH. Anti-Cancer Agents Med. Chem..

[96] Morais M., Oliveira B.L., Correia J.D.G., Oliveira M.C., Jimenez M.A., Santos I., Raposinho P.D. (2013). Influence of the Bifunctional Chelator on the Pharmacokinetic Properties of Tc-99m(CO)_3_-Labeled Cyclic alpha-Melanocyte Stimulating Hormone Analog. J. Med. Chem..

[97] Raposinho P.D., Correia J.D.G., Oliveira M.C., Santos I. (2010). Melanocortin-1 Receptor-Targeting With Radiolabeled Cyclic alpha-Melanocyte-Stimulating Hormone Analogs for Melanoma Imaging. Biopolymers.

[98] Ren G., Pan Y., Cheng Z. (2010). Molecular Probes for Malignant Melanoma Imaging. Curr. Pharm. Biotechnol..

[99] Quinn T., Zhang X., Miao Y. (2010). Targeted melanoma imaging and therapy with radiolabeled alpha-melanocyte stimulating hormone peptide analogues. G. Ital. Dermatol. Venereol..

[100] Nagy G., Denes N., Kis A., Szabo J.P., Berenyi E., Garai I., Bai P., Hajdu I., Szikra D., Trencsenyi G. (2017). Preclinical evaluation of melanocortin-1 receptor (MC1-R) specific Ga-68-and Sc-44-labeled DOTA-NAPamide in melanoma imaging. Eur. J. Pharm. Sci..

[101] Carta D., Salvarese N., Morellato N., Gao F., Sihver W., Pietzsch H.J., Biondi B., Ruzza P., Refosco F., Carpanese D. (2016). Melanoma targeting with Tc-99m(N)(PNP3)-labeled alpha-melanocyte stimulating hormone peptide analogs: Effects of cyclization on the radiopharmaceutical properties. Nucl. Med. Biol..

[102] Gao F., Sihver W., Jurischka C., Bergmann R., Haase-Kohn C., Mosch B., Steinbach J., Carta D., Bolzati C., Calderan A. (2016). Radiopharmacological characterization of Cu-64-labeled alpha-MSH analogs for potential use in imaging of malignant melanoma. Amino Acids.

[103] Rangger C., Helbok A., Ocak M., Radolf T., Andreae F., Virgolini I.J., Von Guggenberg E., Decristoforo C. (2013). Design and Evaluation of Novel Radiolabelled VIP Derivatives for Tumour Targeting. Anticancer Res..

[104] Tang B., Yong X., Xie R., Li Q.W., Yang S.M. (2014). Vasoactive intestinal peptide receptor- based imaging and treatment of tumors (Review). Int. J. Oncol..

[105] Tripathi S.K., Kumar P., Trabulsi E.J., Kim S., McCue P.A., Intenzo C., Berger A., Gomella L., Thakur M.L. (2017). VPAC1 Targeted Cu-64-TP3805 kit preparation and its evaluation. Nucl. Med. Biol..

[106] Tripathi S., Trabulsi E.J., Gomella L., Kim S., McCue P., Intenzo C., Birbe R., Gandhe A., Kumar P., Thakur M. (2016). VPAC1 Targeted Cu-64-TP3805 Positron Emission Tomography Imaging of Prostate Cancer: Preliminary Evaluation in Man. Urology.

[107] Funke U., Vugts D.J., Janssen B., Spaans A., Kruijer P.S., Lammertsma A.A., Perk L.R., Windhorst A.D. (2013). 11C-labeled and 18F-labeled PET ligands for subtype-specific imaging of histamine receptors in the brain. J. Label. Compd. Radiopharm..

[108] Schou M., Varnas K., Jureus A., Ahlgren C., Malmquist J., Haggkvist J., Tari L., Wesolowski S.S., Throner S.R., Brown D.G. (2016). Discovery and Preclinical Validation of C-11 AZ13153556, a Novel Probe for the Histamine Type 3 Receptor. ACS Chem. Neurosci..

[109] Lewis D.Y., Champion S., Wyper D., Dewar D., Pimlott S. (2014). Assessment of I-125 WYE-230949 as a Novel Histamine H-3 Receptor Radiopharmaceutical. PLoS ONE.

[110] Koga K., Maeda J., Tokunaga M., Hanyu M., Kawamura K., Ohmichi M., Nakamura T., Nagai Y., Seki C., Kimura Y. (2016). Development of TASP0410457 (TASP457), a novel dihydroquinolinone derivative as a PET radioligand for central histamine H-3 receptors. Ejnmmi Res..

[111] Kimura Y., Seki C., Ikoma Y., Ichise M., Kawamura K., Takahata K., Moriguchi S., Nagashima T., Ishii T., Kitamura S. (2016). C-11 TASP457, a novel PET ligand for histamine H-3 receptors in human brain. Eur. J. Nucl. Med. Mol. Imaging.

[112] Hanyu M., Kawamura K., Takei M., Furutsuka K., Shiomi S., Fujishiro T., Ogawa M., Nengaki N., Hashimoto H., Fukumura T. (2016). Radiosynthesis and quality control of C-11 TASP457 as a clinically useful PET ligand for imaging of histamine H-3 receptors in human brain. Nucl. Med. Biol..

[113] Nebel N., Maschauer S., Kuwert T., Hocke C., Prante O. (2016). In Vitro and In Vivo Characterization of Selected Fluorine-18 Labeled Radioligands for PET Imaging of the Dopamine D3 Receptor. Molecules.

[114] Prante O., Maschauer S., Banerjee A. (2013). Radioligands for the dopamine receptor subtypes. J. Label. Compd. Radiopharm..

[115] Mach R.H. (2017). Small Molecule Receptor Ligands for PET Studies of the Central Nervous System-Focus on G Protein Coupled Receptors. Semin. Nucl. Med..

[116] Banerjee A., Prante O. (2012). Subtype-Selective Dopamine Receptor Radioligands for PET Imaging: Current Status and Recent Developments. Curr. Med. Chem..

[117] Saulin A., Savli M., Lanzenberger R. (2012). Serotonin and molecular neuroimaging in humans using PET. Amino Acids.

[118] Paterson L.M., Kornum B.R., Nutt D.J., Pike V.W., Knudsen G.M. (2013). 5-HT radioligands for human brain imaging with PET and SPECT. Med. Res. Rev..

[119] Hazari P.P., Pandey A., Chaturvedi S., Mishra A.K. (2017). New Trends and Current Status of Positron-Emission Tomography and Single-Photon-Emission Computerized Tomography Radioligands for Neuronal Serotonin Receptors and Serotonin Transporter. Bioconj. Chem..

[120] Tyacke R.J., Nutt D.J. (2015). Optimising PET approaches to measuring 5-HT release in human brain. Synapse.

[121] Hargreaves R.J., Rabiner E.A. (2014). Translational PET imaging research. Neurobiol. Dis..

[122] Honer M., Gobbi L., Martarello L., Comley R.A. (2014). Radiologand development for molecular imaging of the central nervous system with positron emission tomography. Drug Discov. Today.

[123] Gunn R.N., Rabiner E.A. (2017). Imaging in Central Nervous System Drug Discovery. Semin. Nucl. Med..

[124] Declercq L.D., Vandenberghe R., Van Laere K., Verbruggen A., Bormans G. (2016). Drug Development in Alzheimer’s Disease: The Contribution of PET and SPECT. Front. Pharmacol..

[125] Buiter H.J.C., Windhorst A.D., Huisman M.C., Yaqub M., Knol D.L., Fisher A., Lammertsma A.A., Leysen J.E. (2013). C-11 AF150(S), an agonist PET ligand for M1 muscarinic acetylcholine receptors. Ejnmmi Res..

[126] Ravasi L., Tokugawa J., Nakayama T., Seidel J., Sokoloff L., Eckelman W.C., Kiesewetter D.O. (2012). Imaging of the muscarinic acetylcholine neuroreceptor in rats with the M2 selective agonist F-18 FP-TZTP. Nucl. Med. Biol..

[127] Moustapha M.E., Motaleb M.A., Ibrahim I.T. (2011). Synthesis of Tc-99m-oxybutynin for M-3-receptor-mediated imaging of urinary bladder. J. Radioanal. Nucl. Chem..

[128] van Oosten E.M., Wilson A.A., Mamo D.C., Pollock B.G., Mulsant B.H., Houle S., Vasdev N. (2010). Towards the development of new subtype-specific muscarinic receptor radiopharmaceuticals—Radiosynthesis and ex vivo biodistribution of F-18 3-(4-(2-(2-(2-fluoroethoxy)ethoxy)ethylthio)-1,2,5-thiadiazol-3-yl)-1-methyl-1,2,5,6-tetrahydropyridine. Can. J. Chem.-Revue Can. Chim..

[129] Renard P.Y., Jean L. (2017). Probing the cholinergic system to understand neurodegenerative diseases. Futur. Med. Chem..

[130] Radeke H.S., Purohit A., Harris T.D., Hanson K., Jones R., Hu C., Yalamanchili P., Hayes M., Yu M., Guaraldi M. (2011). Synthesis and Cardiac Imaging of F-18-Ligands Selective for beta(1)-Adrenoreceptors. ACS Med. Chem. Lett..

[131] Kopka K., Law M.P., Breyholz H.J., Faust A., Holtke C., Riemann B., Schober O., Schafers M., Wagner S. (2005). Non-invasive molecular imaging of beta-adrenoceptors in vivo: Perspectives for PET-radioligands. Curr. Med. Chem..

[132] Wollenweber T., Bengel F.M. (2014). Cardiac Molecular Imaging. Semin. Nucl. Med..

[133] Lehto J., Virta J.R., Oikonen V., Roivainen A., Luoto P., Arponen E., Helin S., Hietamaki J., Holopainen A., Kailajarvi M. (2015). Test-retest reliability of C-11-ORM-13070 in PET imaging of alpha(2C)-adrenoceptors in vivo in the human brain. Eur. J. Nucl. Med. Mol. Imaging.

[134] Luoto P., Suilamo S., Oikonen V., Arponen E., Helin S., Herttuainen J., Hietamaki J., Holopainen A., Kailajarvi M., Peltonen J.M. (2014). C-11-ORM-13070, a novel PET ligand for brain alpha(2C)-adrenoceptors: Radiometabolism, plasma pharmacokinetics, whole-body distribution and radiation dosimetry in healthy men. Eur. J. Nucl. Med. Mol. Imaging.

[135] Arponen E., Helin S., Marjamaki P., Gronroos T., Holm P., Loyttyniemi E., Nagren K., Scheinin M., Haaparanta-Solin M., Sallinen J. (2014). A PET Tracer for Brain alpha(2C) Adrenoceptors, C-11-ORM-13070: Radiosynthesis and Preclinical Evaluation in Rats and Knockout Mice. J. Nucl. Med..

[136] Evens N., Bormans G.M. (2010). Non-Invasive Imaging of the Type 2 Cannabinoid Receptor, Focus on Positron Emission Tomography. Curr. Top. Med. Chem..

[137] Horti A.G., Van Laere K. (2008). Development of Radioligands for In Vivo Imaging of Type 1 Cannabinoid Receptors (CB1) in Human Brain. Curr. Pharm. Des..

[138] Navarro G., Morales P., Rodriguez-Cueto C., Fernandez-Ruiz J., Jagerovic N., Franco R. (2016). Targeting Cannabinoid CB2 Receptors in the Central Nervous System. Medicinal Chemistry Approaches with Focus on Neurodegenerative Disorders. Front. Neurosci..

[139] Moldovan R.P., Teodoro R., Gao Y.J., Deuther-Conrad W., Kranz M., Wang Y.C., Kuwabara H., Nakano M., Valentine H., Fischer S. (2016). Development of a High-Affinity PET Radioligand for Imaging Cannabinoid Subtype 2 Receptor. J. Med. Chem..

[140] Caille F., Cacheux F., Peyronneau M.A., Jego B., Jaumain E., Pottier G., Ullmer C., Grether U., Winkeler A., Dolle F. (2017). From Structure-Activity Relationships on Thiazole Derivatives to the In Vivo Evaluation of a New Radiotracer for Cannabinoid Subtype 2 PET Imaging. Mol. Pharm..

[141] Moldovan R.P., Hausmann K., Deuther-Conrad W., Brust P. (2017). Development of Highly Affine and Selective Fluorinated Cannabinoid Type 2 Receptor Ligands. ACS Med. Chem. Lett..

[142] Ahamed M., van Veghel D., Ullmer C., Van Laere K., Verbruggen A., Bormans G.M. (2016). Synthesis, Biodistribution and in vitro Evaluation of Brain Permeable High Affinity Type 2 Cannabinoid Receptor Agonists C-11 MA2 and F-18 MA3. Front. Neurosci..

[143] Vuorimaa A., Rissanen E., Airas L. (2017). In Vivo PET Imaging of Adenosine 2A Receptors in Neuroinflammatory and Neurodegenerative Disease. Contrast Media Mol. Imaging.

[144] Mishina M., Ishiwata K. (2014). Adenosine Receptor PET Imaging in Human Brain. Adenosine Recept. Neurol. Psychiatr..

[145] van Waarde A., Dierckx R., Zhou X.Y., Khanapur S., Tsukada H., Ishiwata K., Luurtsema G., de Vries E.F.J., Elsinga P.H. (2018). Potential Therapeutic Applications of Adenosine A(2A) Receptor Ligands and Opportunities for A(2A) Receptor Imaging. Med. Res. Rev..

[146] Albrecht D.S., Granziera C., Hooker J.M., Loggia M.L. (2016). In Vivo Imaging of Human Neuroinflammation. ACS Chem. Neurosci..

[147] Masino S.A., Kawamura M., Ruskin D.N., Mori A. (2014). Adenosine Receptors and Epilepsy: Current Evidence and Future Potential. Adenosine Receptors in Neurology and Psychiatry.

[148] Gruber S., Ametamey S.M. (2017). Imaging the glutamate receptor subtypes-Much achieved, and still much to do. Drug Discov. Today Technol..

[149] Majo V.J., Prabhakaran J., Mann J.J., Kumar J.S.D. (2013). PET and SPECT tracers for glutamate receptors. Drug Discov. Today.

[150] Li S.Y., Huang Y.Y. (2014). In Vivo Imaging of the Metabotropic Glutamate Receptor 1 (mGluR1) with Positron Emission Tomography: Recent Advance and Perspective. Curr. Med. Chem..

[151] Kuil J., Buckle T., van Leeuwen F.W.B. (2012). Imaging agents for the chemokine receptor 4 (CXCR4). Chem. Soc. Rev..

[152] Bluemel C., Hahner S., Heinze B., Fassnacht M., Kroiss M., Bley T.A., Wester H.J., Kropf S., Lapa C., Schirbel A. (2017). Investigating the Chemokine Receptor 4 as Potential Theranostic Target in Adrenocortical Cancer Patients. Clin. Nucl. Med..

[153] Buck A.K., Stolzenburg A., Hanscheid H., Schirbel A., Luckerath K., Schottelius M., Wester H.J., Lapa C. (2017). Chemokine receptor—Directed imaging and therapy. Methods.

[154] Lapa C., Kircher S., Schirbel A., Rosenwald A., Kropf S., Pelzer T., Walles T., Buck A.K., Weber W.A., Wester H.J. (2017). Targeting CXCR4 with 68Ga Pentixafor: A suitable theranostic approach in pleural mesothelioma. Oncotarget.

[155] Schwarzenbok S.M., Stenzel J., Otto T., Helldorff H.V., Bergner C., Kurth J., Polei S., Lindner T., Rauer R., Hohn A. (2017). Ga-68 pentixafor for CXCR4 imaging in a PC-3 prostate cancer xenograft model—Comparison with F-18 FDG PET/CT, MRI and ex vivo receptor expression. Oncotarget.

[156] Walenkamp A.M.E., Lapa C., Herrmann K., Wester H.J. (2017). CXCR4 Ligands: The Next Big Hit?. J. Nucl. Med..

[157] Watts A., Singh B., Basher R., Singh H., Bal A., Kapoor R., Arora S.K., Wester H.J., Mittal B.R., Behera D. (2017). 68Ga-Pentixafor PET/CT demonstrating higher CXCR4 density in small cell lung carcinoma than in non-small cell variant. Eur. J. Nucl. Med. Mol. Imaging.

[158] Werner R.A., Weich A., Higuchi T., Schmid J.S., Schirbel A., Lassmann M., Wild V., Rudelius M., Kudlich T., Herrmann K. (2017). Imaging of Chemokine Receptor 4 Expression in Neuroendocrine Tumors—A Triple Tracer Comparative Approach. Theranostics.

[159] Habringer S., Herhaus P., Schottelius M., Lapa C., Istvanffy R., Gotze K., Steiger K., Vick B., Peschel C., Oostendorp R. (2016). Peptide-Receptor Radiotherapy with CXCR4-Targeting Pentixather Reduces Leukemia Burden in Acute Leukemia PDX and Patients. Blood.

[160] Schulte G. (2010). International Union of Basic and Clinical Pharmacology. LXXX. The Class Frizzled Receptors. Pharmacol. Rev..

[161] Zeng C.M., Chen Z., Fu L. (2018). Frizzled Receptors as Potential Therapeutic Targets in Human Cancers. Int. J. Mol. Sci..

[162] Schulte G., Wright S.C. (2018). Frizzleds as GPCRs—More Conventional Than We Thought!. Trends Pharmacol. Sci..

[163] Nagayama S., Katagiri T., Tsunoda T., Hosaka T., Nakashima Y., Araki N., Kusuzaki K., Nakayama T., Tsuboyama T., Nakamura T. (2002). Genome-wide analysis of gene expression in synovial sarcomas using a cDNA microarray. Cancer Res..

[164] Fukukawa C., Hanaoka H., Nagayama S., Tsunoda T., Toguchida J., Endo K., Nakamura Y., Katagiri T. (2008). Radioimmunotherapy of human synovial sarcoma using a monoclonal antibody against FZD10. Cancer Sci..

[165] Hanaoka H., Katagiri T., Fukukawa C., Yoshioka H., Yamamoto S., Iida Y., Higuchi T., Oriuchi N., Paudyal B., Paudyal P. (2009). Radioimmunotherapy of solid tumors targeting a cell-surface protein, FZD10: Therapeutic efficacy largely depends on radiosensitivity. Ann. Nucl. Med..

[166] Giraudet A.-L., Cassier P.A., Iwao-Fukukawa C., Garin G., Badel J.-N., Kryza D., Chabaud S., Gilles-Afchain L., Clapisson G., Desuzinges C. (2018). A first-in-human study investigating biodistribution, safety and recommended dose of a new radiolabeled MAb targeting FZD10 in metastatic synovial sarcoma patients. BMC Cancer.

[167] Li H.K., Sugyo A., Tsuji A.B., Morokoshi Y., Minegishi K., Nagatsu K., Kanda H., Harada Y., Nagayama S., Katagiri T. (2018). alpha-particle therapy for synovial sarcoma in the mouse using an astatine-211-labeled antibody against frizzled homolog 10. Cancer Sci..

[168] Lindegren S., Frost S., Baeck T., Haglund E., Elgvist J., Jensen H. (2008). Direct procedure for the production of At-211-labeled antibodies with an epsilon-lysyl-3-(trimethylstannyl)benzamide immunoconjugate. J. Nucl. Med..

[169] Zhang W., Lu W.Y., Ananthan S., Suto M.J., Li Y.H. (2017). Discovery of novel frizzled-7 inhibitors by targeting the receptor’s transmembrane domain. Oncotarget.

[170] Janda C.Y., Waghray D., Levin A.M., Thomas C., Garcia K.C. (2012). Structural Basis of Wnt Recognition by Frizzled. Science.

[171] Dann C.E., Hsieh J.C., Rattner A., Sharma D., Nathans J., Leahy D.J. (2001). Insights into Wnt binding and signalling from the structures of two Frizzled cysteine-rich domains. Nature.

[172] Lee H.J., Bao J., Miller A., Zhang C., Wu J.B., Baday Y.C., Guibao C., Li L., Wu D.Q., Zheng J.J. (2015). Structure-based Discovery of Novel Small Molecule Wnt Signaling Inhibitors by Targeting the Cysteine-rich Domain of Frizzled. J. Biol. Chem..

[173] Davenport A.P., Bonner T.I., Foord S.M., Harmar A.J., Neubig R.R., Pin J.P., Spedding M., Kojima M., Kangawa K. (2005). International Union of Pharmacology. LVI. Ghrelin receptor nomenclature, distribution, and function. Pharmacol. Rev..

[174] Bodart V., Febbraio M., Demers A., McNicoll N., Pohankova P., Perreault A., Sejlitz T., Escher E., Silverstein R.L., Lamontagne D. (2002). CD36 mediates the cardiovascular action of growth hormone-releasing peptides in the heart. Circ. Res..

[175] Muccioli G., Pons N., Ghe C., Catapano F., Granata R., Ghigo E. (2004). Ghrelin and des-acyl ghrelin both inhibit isoproterenol-induced lipolysis in rat adipocytes via a non-type 1a growth hormone secretagogue receptor. Eur. J. Pharmacol..

[176] Delhanty P.J.D., van der Eerden B.C.J., van der Velde M., Gauna C., Pols H.A.P., Jahr H., Chiba H., van der Lely A.J., van Leeuwen J. (2006). Ghrelin and unacylated ghrelin stimulate human osteoblast growth via mitogen-activated protein kinase (MAPK)/phosphoinositide 3-kinase (PI3K) pathways in the absence of GHS-R1a. J. Endocrinol..

[177] Thompson N.M., Gill D.A.S., Davies R., Loveridge N., Houston P.A., Robinson I., Wells T. (2004). Ghrelin and des-octanoyl ghrelin promote adipogenesis directly in vivo by a mechanism independent of the type 1a growth hormone secretagogue receptor. Endocrinology.

[178] Cruz C.R.Y., Smith R.G., Litwack G. (2008). The growth hormone secretagogue receptor. Vitamins and Hormones, Ghrelin.

[179] Bednarek M.A., Feighner S.D., Pong S.S., McKee K.K., Hreniuk D.L., Silva M.V., Warren V.A., Howard A.D., Van der Ploeg L.H.Y., Heck J.V. (2000). Structure-function studies on the new growth hormone-releasing peptide, ghrelin: Minimal sequence of ghrelin necessary for activation of growth hormone secretagogue receptor 1a. J. Med. Chem..

[180] Mani B.K., Zigman J.M. (2017). Ghrelin as a Survival Hormone. Trends Endocrinol. Metab..

[181] Lin T.C., Hsiao M. (2017). Ghrelin and cancer progression. Biochim. Biophys. Acta-Rev. Cancer.

[182] Beiras-Fernandez A., Kreth S., Weis F., Ledderose C., Pottinger T., Dieguez C., Beiras A., Reichart B. (2010). Altered myocardial expression of ghrelin and its receptor (GHSR-1a) in patients with severe heart failure. Peptides.

[183] Katugampola S.D., Pallikaros Z., Davenport A.P. (2001). I-125-His(9) -Ghrelin, a novel radioligand for localizing GHS orphan receptors in human and rat tissue; up-regulation of receptors with atherosclerosis. Br. J. Pharmacol..

[184] Muccioli G., Papotti M., Locatelli V., Ghigo E., Deghenghi R. (2001). Binding of I-125-labeled ghrelin to membranes from human hypothalamus and pituitary gland. J. Endocrinol. Investig..

[185] Harrold J.A., Dovey T., Cai X.J., Halford J.C.G., Pinkney J. (2008). Autoradiographic analysis of ghrelin receptors in the rat hypothalamus. Brain Res..

[186] Wojciuk G., Kruszewski M. (2018). DTPA-(PABn)-Leu(5)-des-acyl ghrelin(1-5) as a new carrier of radionuclides and potential precursor of radiopharmaceuticals. Nucl. Med. Commun..

[187] Kozminski P., Gniazdowska E. (2015). Synthesis and in vitro/in vivo evaluation of novel mono- and trivalent technetium-99m labeled ghrelin peptide complexes as potential diagnostic radiopharmaceuticals. Nucl. Med. Biol..

[188] Chollet C., Bergmann R., Pietzsch J., Beck-Sickinger A.G. (2012). Design, Evaluation, and Comparison of Ghrelin Receptor Agonists and Inverse Agonists as Suitable Radiotracers for PET Imaging. Bioconj. Chem..

[189] Carlie L.C., Savita D., Leonard G.L. (2014). Evaluation of Ga-68-DOTA ghrelin (1-19) in LNCaP prostate carcinoma. Nucl. Med. Biol..

[190] Charron C.L., Hou J.N., McFarland M.S., Dhanvantari S., Kovacs M.S., Luyt L.G. (2017). Structure Activity Study of Ghrelin(1-8) Resulting in High Affinity Fluorine-Bearing Ligands for the Ghrelin Receptor. J. Med. Chem..

[191] Morris G.M., Huey R., Lindstrom W., Sanner M.F., Belew R.K., Goodsell D.S., Olson A.J. (2009). AutoDock4 and AutoDockTools4: Automated Docking with Selective Receptor Flexibility. J. Comput. Chem..

[192] Holst B., Frimurer T.M., Mokrosinski J., Halkjaer T., Cullberg K.B., Underwood C.R., Schwartz T.W. (2009). Overlapping Binding Site for the Endogenous Agonist, Small-Molecule Agonists, and Ago-allosteric Modulators on the Ghrelin Receptor. Mol. Pharmacol..

[193] McGirr R., McFarland M.S., McTavish J., Luyt L.G., Dhanvantari S. (2011). Design and characterization of a fluorescent ghrelin analog for imaging the growth hormone secretagogue receptor 1a. Regul. Pept..

[194] Lu C., McFarland M.S., Nesbitt R.L., Williams A.K., Chan S., Gomez-Lemus J., Autran-Gomez A.M., Al-Zahrani A., Chin J.L., Izawa J.I. (2012). Ghrelin receptor as a novel imaging target for prostatic neoplasms. Prostate.

[195] Douglas G.A.F., McGirr R., Charlton C.L., Kagan D.B., Hoffman L.M., Luyt L.G., Dhanvantari S. (2014). Characterization of a far-red analog of ghrelin for imaging GHS-R in P19-derived cardiomyocytes. Peptides.

[196] Rosita D., Dewit M.A., Luyt L.G. (2009). Fluorine and Rhenium Substituted Ghrelin Analogues as Potential Imaging Probes for the Growth Hormone Secretagogue Receptor. J. Med. Chem..

[197] Potter R., Horti A.G., Ravert H.T., Holt D.P., Finley P., Scheffel U., Dannals R.F., Wahl R.L. (2011). Synthesis and in vivo evaluation of (S)-6-(4-fluorophenoxy)-3-((1-C-11 methylpiperidin-3-yl)methyl)-2-*o*-tol ylquinazolin-4(3H)-one, a potential PET tracer for growth hormone secretagogue receptor (GHSR). Bioorg. Med. Chem..

[198] Kawamura K., Fujinaga M., Shimoda Y., Yamasaki T., Zhang Y.D., Hatori A., Xie L., Wakizaka H., Kumata K., Ohkubo T. (2017). Developing new PET tracers to image the growth hormone secretagogue receptor 1a (GHS-R1a). Nucl. Med. Biol..

[199] Hou J.Q., Kovacs M.S., Dhanvantari S., Luyt L.G. (2018). Development of Candidates for Positron Emission Tomography (PET) Imaging of Ghrelin Receptor in Disease: Design, Synthesis, and Evaluation of Fluorine-Bearing Quinazolinone Derivatives. J. Med. Chem..

[200] Barton M., Filardo E.J., Lolait S.J., Thomas P., Maggiolini M., Prossnitz E.R. (2018). Twenty years of the G protein-coupled estrogen receptor GPER: Historical and personal perspectives. J. Steroid Biochem. Mol. Biol..

[201] Prossnitz E.R., Arterburn J.B. (2015). International Union of Basic and Clinical Pharmacology. XCVII. G Protein-Coupled Estrogen Receptor and Its Pharmacologic Modulators. Pharmacol. Rev..

[202] Revankar C.M., Cimino D.F., Sklar L.A., Arterburn J.B., Prossnitz E.R. (2005). A transmembrane intracellular estrogen receptor mediates rapid cell signaling. Science.

[203] Kiyono Y., Mori T., Okazawa H. (2012). Positron Emission Tomography Radiopharmaceuticals for Sex Steroid Hormone Receptor Imaging. Curr. Med. Chem..

[204] Talbot J.N., Gligorov J., Nataf V., Montravers F., Huchet V., Michaud L., Ohnona J., Balogova S., Cussenot O., Darai E. (2015). Current applications of PET imaging of sex hormone receptors with a fluorinated analogue of estradiol or of testosterone. Q. J. Nucl. Med. Mol. Imaging.

[205] van Kruchten M., de Vries E.G.E., Brown M., de Vries E.F.J., Glaudemans A., Dierckx R., Schroder C.P., Hospers G.A.P. (2013). PET imaging of oestrogen receptors in patients with breast cancer. Lancet Oncol..

[206] Ramesh C., Bryant B., Nayak T., Revankar C.M., Anderson T., Carlson K.E., Katzenellenbogen J.A., Sklar L.A., Norenberg J.P., Prossnitz E.R. (2006). Linkage effects on binding affinity and activation of GPR30 and estrogen receptors ER alpha/beta with tridentate pyridin-2-yl hydrazine tricarbonyl-Re/Tc-99m(I) chelates. J. Am. Chem. Soc..

[207] Nayak T.K., Hathaway H.J., Ramesh C., Arterburn J.B., Dai D., Sklar L.A., Norenberg J.P., Prossnitz E.R. (2008). Preclinical development of a neutral, estrogen receptor-targeted, tridentate Tc-99m(I)-estradiol-pyridin-2-yl hydrazine derivative for imaging of breast and endometrial cancers. J. Nucl. Med..

[208] Bologa C.G., Revankar C.M., Young S.M., Edwards B.S., Arterburn J.B., Kiselyov A.S., Parker M.A., Tkachenko S.E., Savchuck N.P., Sklar L.A. (2006). Virtual and biomolecular screening converge on a selective agonist for GPR30. Nat. Chem. Biol..

[209] Dennis M.K., Burai R., Ramesh C., Petrie W.K., Alcon S.N., Nayak T.K., Bologa C.G., Leitao A., Brailoiu E., Deliu E. (2009). In vivo effects of a GPR30 antagonist. Nat. Chem. Biol..

[210] Nayak T.K., Dennis M.K., Ramesh C., Burai R., Atcher R.W., Sklar L.A., Norenberg J.P., Hathaway H.J., Arterburn J.B., Prossnitz E.R. (2010). Influence of Charge on Cell Permeability and Tumor Imaging of GPR30-Targeted In-111-Labeled Nonsteroidal Imaging Agents. ACS Chem. Biol..

[211] Ramesh C., Nayak T.K., Burai R., Dennis M.K., Hathaway H.J., Sklar L.A., Prossnitz E.R., Arterburn J.B. (2010). Synthesis and Characterization of Iodinated Tetrahydroquinolines Targeting the G Protein-Coupled Estrogen Receptor GPR30. J. Med. Chem..

[212] Burai R., Ramesh C., Nayak T.K., Dennis M.K., Bryant B.K., Prossnitz E.R., Arterburn J.B. (2012). Synthesis and Characterization of Tricarbonyl-Re/Tc(I) Chelate Probes Targeting the G Protein-Coupled Estrogen Receptor GPER/GPR30. PLoS ONE.

[213] Nayak T.K., Ramesh C., Hathaway H.J., Norenberg J.P., Arterburn J.B., Prossnitz E.R. (2014). GPER-Targeted, Tc-99m-Labeled, Nonsteroidal Ligands Demonstrate Selective Tumor Imaging and In Vivo Estrogen Binding. Mol. Cancer Res..

[214] Papalia T., Lappano R., Barattucci A., Pisano A., Bruno G., Santolla M.F., Campagna S., De Marco P., Puntoriero F., De Francesco E.M. (2015). A Bodipy as a luminescent probe for detection of the G protein estrogen receptor (GPER). Org. Biomol. Chem..

[215] Brinkmann V., Billich A., Baumruker T., Heining P., Schmouder R., Francis G., Aradhye S., Burtin P. (2010). Fingolimod (FTY720): Discovery and development of an oral drug to treat multiple sclerosis. Nat. Rev. Drug Discov..

[216] Rosenberg A.J., Liu H., Jin H.J., Yue X.Y., Riley S., Brown S.J., Tu Z.D. (2016). Design, Synthesis, and In Vitro and In Vivo Evaluation of an F-18-Labeled Sphingosine 1-Phosphate Receptor 1 (S1P(1)) PET Tracer. J. Med. Chem..

[217] Briard E., Orain D., Beerli C., Billich A., Streiff M., Bigaud M., Auberson Y.P. (2011). BZM055, an Iodinated Radiotracer Candidate for PET and SPECT Imaging of Myelin and FTY720 Brain Distribution. ChemMedChem.

[218] Shaikh R.S., Schilson S.S., Wagner S., Hermann S., Keul P., Levkau B., Schafers M., Haufe G. (2015). Synthesis and Evaluation of Fluorinated Fingolimod (FTY720) Analogues for Sphingosine-1-Phosphate Receptor Molecular Imaging by Positron Emission Tomography. J. Med. Chem..

[219] Prasad V.P., Wagner S., Keul P., Hermann S., Levkau B., Schafers M., Haufe G. (2014). Synthesis of fluorinated analogues of sphingosine-1-phosphate antagonists as potential radiotracers for molecular imaging using positron emission tomography. Bioorg. Med. Chem..

[220] Jin H.J., Yang H., Liu H., Zhang Y.X., Zhang X., Rosenberg A.J., Liu Y.J., Lapi S.E., Tu Z.D. (2017). A promising carbon-11-labeled sphingosine-1-phosphate receptor 1-specific PET tracer for imaging vascular injury. J. Nucl. Cardiol..

[221] Liu H., Jin H.J., Yue X.Y., Luo Z.H., Liu C.L., Rosenberg A.J., Tu Z.D. (2016). PET Imaging Study of S1PR1 Expression in a Rat Model of Multiple Sclerosis. Mol. Imaging Biol..

[222] Liu H., Jin H.J., Yue X.Y., Han J.B., Baum P., Abendschein D.R., Tu Z.D. (2017). PET Study of Sphingosine-1-Phosphate Receptor 1 Expression in Response to Vascular Inflammation in a Rat Model of Carotid Injury. Mol. Imaging.

[223] Liu H., Jin H.J., Han J.B., Yue X.Y., Yang H., Zayed M.A., Gropler R.J., Tu Z.D. (2018). Upregulated Sphingosine 1-Phosphate Receptor 1 Expression in Human and Murine Atherosclerotic Plaques. Mol. Imaging Biol..

[224] Luo Z.H., Rosenberg A.J., Liu H., Han J.B., Tu Z.D. (2018). Syntheses and in vitro evaluation of new S1PR1 compounds and initial evaluation of a lead F-18 radiotracer in rodents. Eur. J. Med. Chem..

[225] Kappos L., Bar-Or A., Cree B.A.C., Fox R.J., Giovannoni G., Gold R., Vermersch P., Arnold D.L., Arnould S., Scherz T. (2018). Siponimod versus placebo in secondary progressive multiple sclerosis (EXPAND): A double-blind, randomised, phase 3 study. Lancet.

[226] Briard E., Rudolph B., Desrayaud S., Krauser J.A., Auberson Y.P. (2015). MS565: A SPECT Tracer for Evaluating the Brain Penetration of BAF312 (Siponimod). ChemMedChem.

[227] Tavares A., Barret O., Alagille D., Morley T., Papin C., Maguire R.P., Briard E., Auberson Y.P., Tamagnan G. (2014). Brain distribution of MS565, an imaging analogue of siponimod (BAF312), in non-human primates. J. Neurol..

[228] Bhattacharya S.K., Cameron K.O.k., Fernando D.P., Kung D.W.-S., Londregan A.T., Mcclure K.F., Simila S.T.M. (2012). 2,3-Dihydro-1H-inden-1-yl-2,7-diazaspiro[3.6] Nonane and Their Use as Antagonists or Inverse Agonists of the Ghrelin Receptor. WO Application.

[229] Luyt L.G., Fowkes M.M. (2018). Peptidomimetics for Imaging the Ghrelin Receptor. U.S. Patent.

